# Interfacial redox mediation by amine/alkyne-functionalized silicon nanoparticles: surfactant-free gold nanoparticle synthesis

**DOI:** 10.1039/d6na00512h

**Published:** 2026-07-01

**Authors:** Amber L. Garcia, Brittany Griggs, Brian S. Mitchell, Mark J. Fink, Julie P. Vanegas

**Affiliations:** a College of Engineering and Computer Science, The University of Texas Rio Grande Valley Edinburg TX 78539 USA; b School of Integrative Biological and Chemical Sciences, The University of Texas Rio Grande Valley Edinburg TX 78539 USA; c Department of Chemical and Biomolecular Engineering, Tulane University New Orleans LA 70118 USA; d Department of Chemistry, Tulane University New Orleans LA 70118 USA; e Department of Physics and Astronomy, The University of Texas Rio Grande Valley Edinburg TX 78539 USA julie.vanegas@utrgv.edu

## Abstract

We develop a surfactant-free synthesis method for gold nanoparticles (AuNPs) using 2-propynylamine-functionalized silicon nanoparticles as integrated redox-active supports. Surface-anchored amine groups act as built-in reducing sites for Au^3+^ precursors at the hexane–water interface under ambient conditions, enabling *in situ* AuNP nucleation and growth. Time-resolved UV-vis spectroscopy and dynamic light scattering track the emergence of plasmonic AuNPs, while quantitative morphology analysis reveals a final population dominated by spherical and near-spherical nanoparticles (∼74%), with faceted polyhedral structures (∼4%) and dendritic, hollow, irregular, and rod-like morphologies (∼22%) as minority species. The temporal evolution proceeds from early dendritic and irregular aggregates to a later-stage ensemble in which small spherical particles dominate numerically, whereas larger faceted structures persist as a minor but visually prominent component. Control experiments with unfunctionalized silicon, amine-functionalized silica, or free ligand alone do not yield AuNPs, underscoring the cooperative role of the semiconductor core and grafted primary amine ligands in mediating interfacial electron transfer. This ligand-enabled strategy provides a scalable route to surfactant-free AuNPs and Au–Si hybrids with potential applications in plasmonic sensing, catalysis, and biomedicine.

## Abstract 2

Interfacial redox mediation by 2-propynylamine-functionalized silicon nanoparticles enables rapid, surfactant-free gold nanoparticle synthesis.

## Graphical abstract

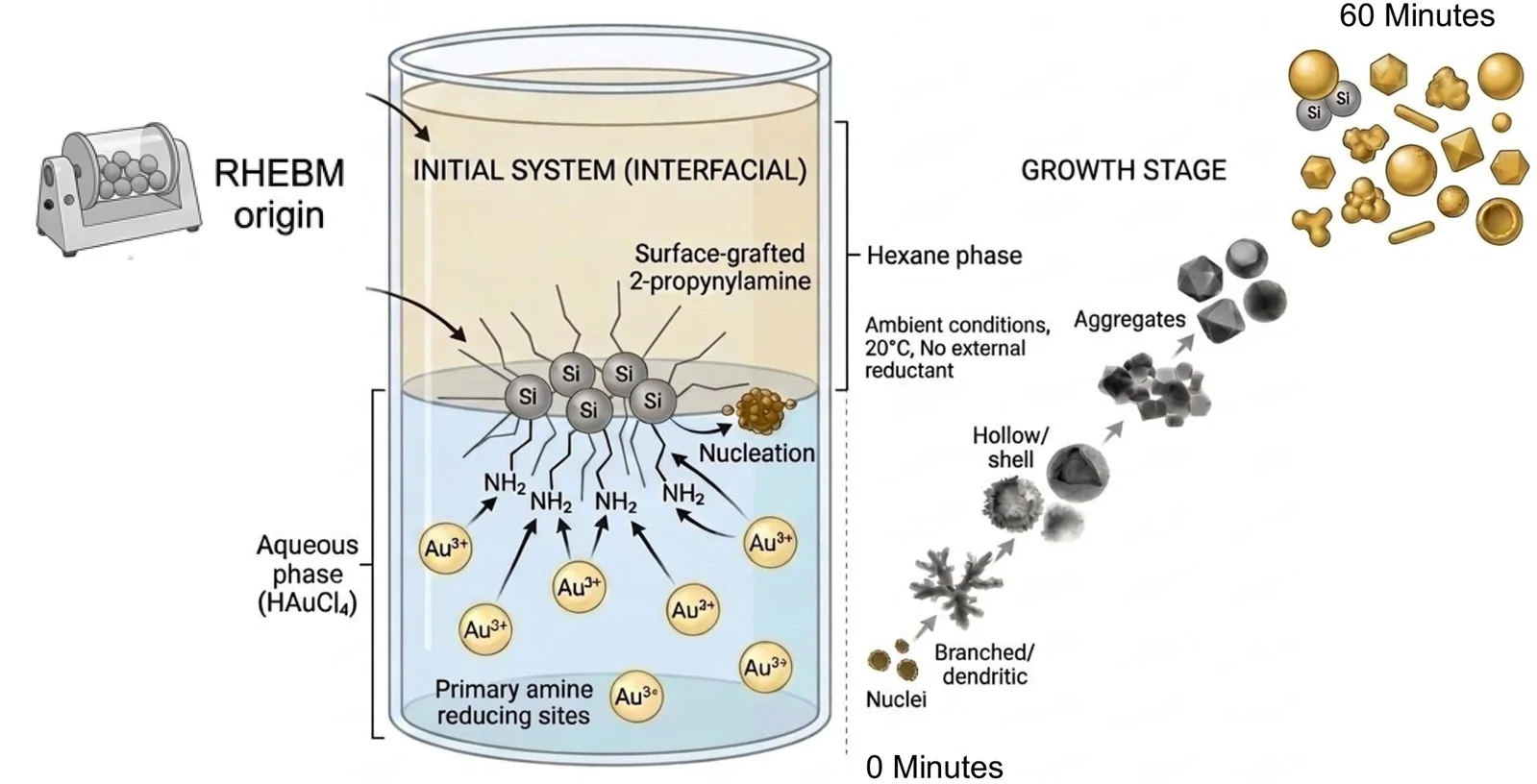

## Introduction

The ability to precisely control the size, shape, and surface chemistry of nanostructured materials has driven major advances in catalysis, optics, biomedicine, and sensing.^1^ In particular, gold nanoparticles (AuNPs) stand out for their morphology-dependent plasmonic and catalytic properties.^2,3^ Polyhedral AuNPs—including rods, stars, and branched nanostructures—exhibit distinct surface plasmon resonance (SPR) across the visible and near-infrared spectrum, enabling broad applicability in sensing and photothermal therapy.^4,5^

Traditional synthetic methods for polyhedral AuNPs often rely on strong chemical reducing agents and surfactants to control particle shape, which limits scalability and sustainability.^6^ Although greener synthesis routes have been developed, achieving reproducible, scalable synthesis of polyhedral AuNPs in hybrid or solution-based nanoparticle dispersion remains challenging.^7^ Conventional approaches frequently rely on reducing agents dispersed in solution and employ inert silica supports that lack redox activity. In contrast, the strategy adopted in this research covalently grafts the reducing functionality onto the silicon nanoparticle (SiNP) surface; the surface-anchored amine groups—immobilized on a semiconductor support rather than free in solution—serve as the reducing sites. This configuration pairs ligand-based redox chemistry with charge transfer from the SiNP core, integrating both reduction pathways within a single platform.^8–10^

Multifunctional ligands represent a key strategy for replacing external reductants while providing opportunities for post-synthetic functionalization. In this context, 2-propynylamine has proven to be an exceptionally versatile molecule due to its bifunctional structure, which combines a primary amine and a terminal alkyne (NH_2_–CH_2_–C≡C). The amine group can coordinate with and donate electrons to Au^3+^, initiating *in situ* reduction, while the alkyne moiety remains chemically accessible for click conjugation on AuNP surfaces.^11,12^ Previous studies have shown that amine-terminated ligands enhance coordination to Au surfaces and modulate electron density, thereby improving dispersion stability and catalytic performance.^13^ At the same time, terminal alkyne functionalities enable modular bioconjugation *via* click chemistry, offering a straightforward route for surface modification.^14^

Our previous work demonstrated that 2-propynylamine independently reduces Au^3+^ to Au while stabilizing the resulting nanoparticles in an aqueous, solvent-free mechanochemical process, yielding click-ready AuNPs.^11^ This finding established 2-propynylamine as a bifunctional reducing and capping agent, capable of producing monodisperse AuNPs without additional reductants or surfactants.

Our present study extends that concept by introducing amine- and alkyne-functionalized SiNPs as interfacial redox mediators. Unlike the high-energy, mechanochemical approach, synthesis in this study occurs entirely under ambient conditions, eliminating mechanical activation but maintaining intrinsic reduction pathways. Here, SiNPs functionalized with 2-propynylamine (SiNPs@2-propynylamine) facilitate electron transfer and nucleation during AuNP growth, serving as interfacial reducing and nucleation sites rather than structural templates that dictate the final nanoparticle size and shape.

Our SiNP-mediated approach exploits the combined semiconductor and surface-redox activity of functionalized SiNPs. The silicon core promotes interfacial charge transfer, while the surface-anchored amine ligands provide localized electron-donating sites that reduce Au^3+^*in situ*, collectively serving as an integrated reducing and nucleation platform. It integrates the redox chemistry of semiconductor nanoparticles with the bifunctional reactivity of 2-propynylamine to facilitate directed AuNP formation *via* interfacial charge transfer. This work demonstrates a sustainable pathway for polyhedral AuNP synthesis bridging green chemistry and semiconductor-mediated reduction.

## Experimental

### Materials

All reagents were of analytical grade and used as received, without further purification. Single-crystal silicon wafers (single-side polished, undoped, 〈111〉 orientation; mirror-finished surface) were obtained from Sigma-Aldrich (St. Louis, MO, USA). 2-Propynylamine (HC≡CCH_2_NH_2_, 98%) was purchased from Alfa Aesar (Ward Hill, MA, USA). Chloroauric acid trihydrate (HAuCl_4_·3H_2_O, 99.9%) and hexane (anhydrous, ≥95%) were supplied by Sigma-Aldrich (St. Louis, MO, USA). Deionized water was purified using a Milli-Q Direct-Q® 3 UV system (Merck Millipore, Burlington, MA, USA). For Control 3, amine-functionalized silica nanoparticles (SiO_2_ methoxypropylaminosilane), ∼30 nm, surface functionalization; cat. no. 791334) were purchased from Sigma-Aldrich (St. Louis, MO, USA). Silicon powder (≥99%, −325 mesh), used as the micron-scale silicon precursor in Control 4, was obtained from Sigma-Aldrich (St. Louis, MO, USA). 1-Bromododecyne (97%), used for the synthesis of SiNPs@1-bromododecyne employed in Control 5, and *N*-methyl-*N*-benzyl-2-propyn-1-amine hydrochloride (pargyline hydrochloride, ≥99%), used for the synthesis of SiNPs@*N*-methyl-*N*-benzyl-2-propynylamine employed in Control 6, were obtained from Sigma-Aldrich (St. Louis, MO, USA).

### Methods

#### Synthesis of SiNPs@2-propynylamine *via* reactive high-energy ball milling (RHEBM)

Sample preparation was carried out inside a nitrogen-filled glovebox to minimize exposure to oxygen and moisture. Mechanically fractured single-crystal silicon wafers (200 mg, 7.12 mmol) were combined with 2-propynylamine (1.40 mL, 21.1 mmol, 98% purity; *ρ* = 0.854 g mL^−1^, *M* = 55.08 g mol^−1^), at an approximate 1 : 3 molar ratio of silicon atoms to ligand, in a hardened stainless-steel milling vial along with three stainless-steel balls (5 mm diameter, 0.21 g each). Hexane (10 mL) was added as a liquid milling medium to improve homogenization and regulate milling dynamics. All components were combined directly in the vial; no filtration or centrifugation was performed prior to milling. The vial was sealed hand-tight within the glovebox and further secured with a wrench immediately after removal.

The surface chemistry of the silicon source was characterized by high-resolution Si 2p, O 1s, and C 1s XPS at four processing stages (wafer/powder, etched/unetched) prior to RHEBM. The corresponding spectra were fit to a layered core/oxide structural model, from which the native-oxide thickness at each stage was calculated and shown to decrease progressively across the four processing stages (4.92 → 3.64 → 2.38 → 0.62 nm).

RHEMB was performed for 3 h using a SPEX CertiPrep 8000D Mixer/Mill (SPEX SamplePrep, Metuchen, NJ, USA). After milling, the reaction mixture was transferred into sterile 50 mL conical centrifuge tubes (VWR) and centrifuged two times at 3800 rpm for 15 min each time. The resulting pellet, containing the functionalized silicon nanomaterial (SiNPs@2-propynylamine), was retained for subsequent analyses. The supernatant was collected, dried, and analyzed *via* UV-vis spectroscopy to determine the presence of unbound 2-propynylamine.

Synthesis of AuNPs@SiNPs@2-propynylamine in a biphasic hexane–water system. AuNPs functionalized with SiNPs@2-propynylamine (AuNPs@SiNPs@2-propynylamine) were prepared by first dissolving HAuCl_4_·3H_2_O (200 mg, 0.51 mmol of Au) in 100 µL of deionized water. Separately, 15 mg of dried SiNPs@2-propynylamine (0.53 mmol of Si; previously stored in hexane as a yellow, viscous solid) were dispersed in hexane and added dropwise to the aqueous gold solution under gentle stirring, forming a biphasic hexane–water system. This configuration accommodated the poor solubility of SiNPs in water and the limited solubility of gold precursors in nonpolar solvents. To enhance interfacial mixing and maintain phase balance, small aliquots of hexane and deionized water were added incrementally during the reaction. The overall molar ratio of Si to Au was approximately 1-to-1.

The mixture was stirred at 20 °C for 1 h in the absence of light to prevent photo-induced side reactions. Although hexane and water are immiscible, the presence of SiNPs produces strong interfacial mixing. The reaction occurs at the hexane–water interface, where SiNPs reside at the boundary between the phases. Gold precursors in the aqueous phase access the amine-functionalized sites at this interface, enabling interfacial electron transfer and Au nucleation. The small size of the SiNPs is central to this process: their high surface-to-volume ratio and localization at the hexane–water interface maximize contact between the aqueous Au^3+^ precursor and the surface amine sites, promoting interfacial electron transfer and nucleation.

Reaction progress was monitored visually: as the AuNPs nucleated and gradually grew on the functionalized SiNP surface, the color evolved smoothly from light yellow to deep red, as illustrated in the series of scintillation vials in Fig. 2. The continuous color transition confirmed successful formation of AuNP-decorated SiNPs without any phase separation steps.

Control experiments for polyhedral AuNP formation. To systematically evaluate the contribution of each component to interfacial Au^3+^ reduction, six control experiments were performed. In Control 1, silicon powder was reacted with HAuCl_4_·3H_2_O in the absence of any surface ligand. In Control 2, free 2-propynylamine was reacted with HAuCl_4_·3H_2_O in the absence of silicon. Control 3 employed amine-functionalized SiO_2_NPs (SiO_2_methoxypropylaminosilane, ∼30 nm) to isolate the role of surface amine groups from the semiconducting nature of silicon. Control 4 consisted of micron-scale silicon powder functionalized with 2-propynylamine. Control 5 used SiNPs functionalized with a non-amine ligand, 1-bromododecyne (SiNPs@1-bromododecyne). Finally, Control 6 employed SiNPs functionalized with *N*-methyl-*N*-benzyl-2-propynylamine, a tertiary amine lacking a free N–H group. All control reactions were carried out in either the biphasic hexane–water system or in water, as appropriate, under the corresponding reaction conditions used for each experiment. AuNP formation was monitored by the characteristic color change from yellow to red and by the appearance of an SPR band at 520–530 nm in the UV-vis spectra.

#### Spectroscopy and imaging characterization

##### UV-vis spectroscopy

UV-vis absorption spectra were recorded at room temperature with a Varian Cary 50 spectrophotometer (Varian Inc., Palo Alto, CA, USA), using 1 cm quartz cuvettes. Spectra were collected over the 200–800 nm range. A constant-temperature sipper system was used to maintain the sample at a stable temperature during UV-vis measurements. Spectral data were processed and analyzed using OriginPro 2025 (64 bit) SR1, version 10.2.0.196 (OriginLab Corporation, Northampton, MA, USA).^15^

##### Transmission electron microscopy (TEM)

TEM and scanning TEM (STEM) analyses were performed using an FEI Tecnai G2 F30 TWIN transmission electron microscope (FEI Company, Hillsboro, OR, USA) equipped with a field emission gun and operated at an accelerating voltage of 300 kV, providing a TEM point resolution of 0.24 nm, line resolution of 0.144 nm, and information limit of 0.15 nm. The instrument was equipped with HAADF-STEM detection, an SDD-based energy-dispersive X-ray spectroscopy (EDS) system for elemental mapping, and an FEI Eagle 4 k high-sensitivity CCD camera interfaced with the Tecnai user interface. Images were acquired in bright-field TEM mode at nominal magnifications ranging from 500 00× to 2 000 00× with exposure times of 1 to 2 seconds. The 0.24 nm point resolution of this instrument was sufficient to resolve the morphological features reported herein, namely dendritic aggregates, hollow shells, and faceted polyhedral nanoparticles ranging from 10 to 100 nm.

For TEM imaging, SiNPs@2-propynylamine grids were prepared by drop-casting onto carbon-coated, formvar-supported copper grids (200 mesh, 200–300 Å carbon layer) from the stable hexane dispersion, which dries rapidly under ambient conditions and yields uniformly distributed individual nanoparticles. For the AuNPs@SiNPs@2-propynylamine hybrid, two complementary sampling strategies were used: (1) an aliquot was taken from the hexane–water interface of the biphasic reaction mixture, where the colored hybrid product concentrates, and (2) a second aliquot was taken from the homogenized biphasic mixture immediately after gentle mixing, to access representative particles from both phases. Because the aqueous component dominates the resulting droplet in both sampling strategies, hybrid grids were dried under ambient conditions for at least 72 h before imaging to ensure complete solvent evaporation.

Particle size and morphology analysis was performed on TEM images using ImageJ software (version 1.53 k). Independent size information was also obtained by dynamic light scattering (DLS) on aliquots of the reaction mixture. Nanoparticles were classified into seven morphological categories: spherical, near-spherical, polyhedral, dendritic, hollow, irregular/aggregates, and rod-like. The characteristic dimension of each category was determined as follows: spheres by diameter; near-spherical particles by equivalent (projected-area) spherical diameter; polyhedra by maximum Feret diameter; dendritic structures by longest branch length; hollow shells by outer diameter; irregular particles and aggregates by maximum Feret diameter; and rods by length and width (aspect ratio). For comparative analysis, an equivalent spherical diameter (*D*_eq = 2√(area/π)) was calculated from projected areas. A total of 652 individual particles were classified across 12 representative TEM micrographs covering reaction times from 5 to 60 min, using ImageJ-based contour analysis. Distributions are reported as mean ± standard deviation in Fig. S6–S9 and Table S5 of the SI.^16^

##### Fourier-transform infrared (FTIR) spectroscopy

FTIR spectra were collected on a Bruker VERTEX 70 spectrometer equipped with a diamond attenuated total reflectance (ATR) accessory (Bruker Optik GmbH, Ettlingen, Germany). Samples were directly applied to the diamond crystal using a spatula to ensure uniform coverage. Spectra were recorded over the 4000–400 cm^−1^ range at a resolution of 2 cm^−1^. All data were processed and plotted using OriginPro 2025 (64 bit) SR1, version 10.2.0.196 (OriginLab Corporation, Northampton, MA, USA).^15^

##### X-ray photoelectron spectroscopy (XPS)

XPS analyses were carried out using a Thermo-Fisher Scientific Nexsa G2 Surface Analysis System equipped with a focused Al Kα monochromatic X-ray source (1486.6 eV; Thermo Fisher Scientific, Waltham, MA, USA). Powder samples were evenly spread onto square, double-sided carbon tape, and excess material was carefully removed to ensure uniform surface coverage. Survey spectra were acquired using five scans with a 400 µm spot size, standard lens mode, an energy step size of 1.0 eV, and a pass energy of 200 eV. High-resolution spectra were obtained over an analysis area of approximately 200 × 400 µm, using a pass energy of 50 eV and an energy step size of 0.10 eV. Spectral deconvolution and quantitative analysis were performed with CasaXPS software, version 2.3.25PR1.0 (Casa Software Ltd, Teignmouth, Devon, UK), employing mixed Gaussian–Lorentzian peak shapes for each component.^17^

## Results and discussion

### SiNPs@2-propynylamine: synthesis and characterization

**Fig. 1:**
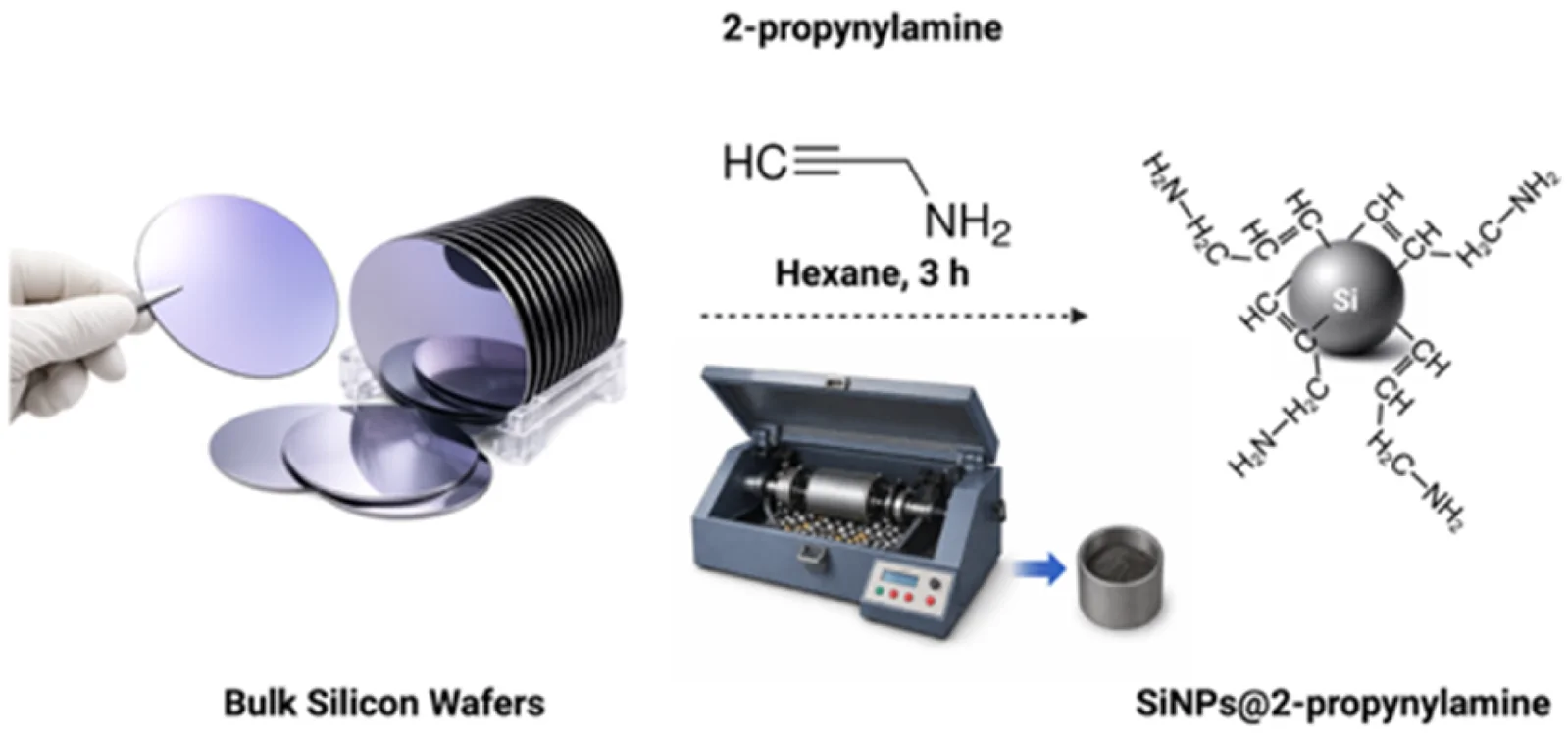
Schematic of the mechanochemical preparation of functionalized SiNPs.

The mechanochemical synthesis of SiNPs@2-propynylamine, used as precursors for the surfactant-free synthesis of AuNPs, is illustrated in Fig. S1, and their characterization is presented in Fig. S2–S4. The functionalized SiNPs dispersed readily in hexane to form a stable yellow suspension. UV-vis spectrum show a distinct absorption band near 300 nm (Fig. S2), characteristic of alkyne-terminated silicon surfaces.^18,19^ The appearance of Si–C and C≡C stretching bands in FTIR confirms successful surface modification (Fig. S4), and TEM images reveal broad size distribution and the partial aggregation typical of mechanochemically prepared SiNPs (Fig. S3). This polydispersity (2–8 nm; mean 3.7 ± 1.1 nm) precludes a systematic evaluation of size-dependent reactivity; future work will address it by fractionating SiNPs into narrow size populations or by tuning milling parameters to obtain monodisperse samples (Fig. 1).

XPS analysis (SI, Fig. S4 and Table S1) provides further insight into the surface chemical states of SiNPs@2-propynylamine. The Si 2p spectrum exhibits components at 99.62 eV (9.10%), 100.25 eV (17.96%), and 101.56 eV (72.94%), corresponding to Si–C or Si^0^, low-valent suboxides (Si^+^/Si^2+^, Si–O–H environments), and higher suboxides (Si^3+^), respectively. The absence of a distinct feature near 103–104 eV confirms that fully oxidized Si^4+^ (SiO_2_) is minimal. In other words, the nanoparticles possess a partially oxidized silicon surface with covalent Si–C bonds to the alkynyl ligand, maintaining both reductive and oxidized domains.^20^

This Si 2p oxidation-state distribution contrasts sharply with that of the silicon source prior to RHEBM. As received, the silicon wafer carries a thick native oxide layer of 4.92 nm (84.92% Si^4+^ at 102.98 eV); mechanical fracturing into powder only reduces this value marginally to 3.64 nm (75.33% Si^4+^). Subsequent chemical etching is markedly more effective on the powder than on the wafer (2.38 nm and 0.62 nm of residual oxide, respectively), reflecting the higher accessible surface area of the powder form. This etched powder, the precursor involved in the RHEBM step, therefore arrives with only ≈0.6 nm of residual oxide and a Si^0^-dominated surface (78.80%; Fig. S5, S13, Tables S2 and S3 of the SI). The complete disappearance of the fully oxidized Si^4+^ component (∼103 eV) in the final SiNPs@2-propynylamine product is consistent with concomitant consumption of residual oxide and grafting of the alkynylamine ligand to the silicon surface during RHEBM.

### Controls

None of the individual components tested (*i.e.*, silicon powder, free ligand, or non-coordinating alkyl-terminated SiNPs) was sufficient to trigger Au^3+^ reduction and AuNP formation on its own under these conditions. The results of the control experiments are summarized in Table S4. Control 1 (no surface ligand) remained pale yellow, with no transition to the characteristic burgundy-red of plasmonic gold and no SPR band near 520 nm. Control 2 (no silicon) likewise showed no optical or plasmonic features. Control 3 (SiO_2_ methoxypropylaminosilane confirmed that surface amine groups alone are insufficient to reduce Au^3+^ and form AuNPs without a semiconductor silicon core. Control 4 (micron-scale Si powder functionalized with 2-propynylamine) did not produce AuNPs, indicating that nanoscale silicon is required. Control 5 (SiNPs@1-bromododecyne) also failed to reduce Au^3+^, suggesting that surface amine functionality is necessary. Control 6 (SiNPs@*N*-methyl-*N*-benzyl-2-propynylamine) produced no SPR band or color change, demonstrating that a primary amine N–H group is required for Au^3+^ coordination and interfacial electron transfer.^8–10^ Together, these results show that both the silicon core and a primary-amine-functionalized surface are required for AuNP formation.

### Evolution (UV-vis and TEM)

**Fig. 2:**
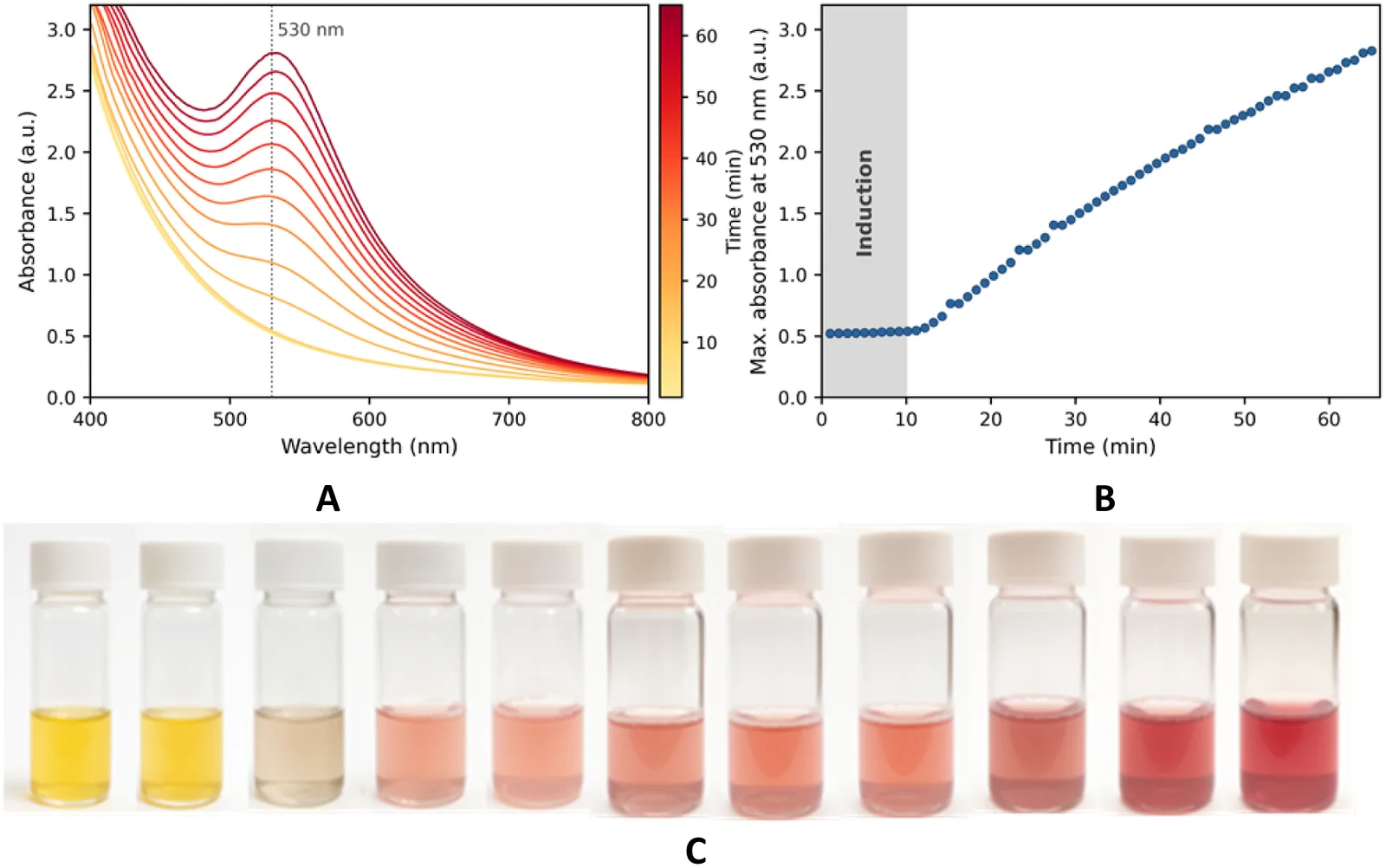
Time-resolved absorption of AuNP formation mediated by SiNPs@2-propynylamine. (A) UV-vis spectra collected over 60 min showing the progressive emergence and growth of the SPR band near 530 nm. (B) Time-dependent increase of maximum absorbance at 530 nm, reflecting the progression of nanoparticle formation. (C) Sequential reaction aliquots collected every 5 min, showing a gradual color change from pale yellow (Au^3+^ precursor) to burgundy-red, confirming the formation of plasmonic AuNPs.

The reduction of Au^3+^ to metallic AuNPs was monitored over 60 minutes using UV-vis spectroscopy. The characteristic absorption band of the Au^3+^ precursor decreases in intensity as the reaction proceeds, accompanied by the appearance of a new SPR band centered near 530 nm, confirming the formation of metallic AuNPs.^21,22^ As shown in Fig. 2A, the SPR band appears within the initial 5 minutes, marking the onset of Au^3+^ reduction and nanoparticle nucleation.^23^ The absorbance then increases roughly linearly over time, indicating a secondary stage dominated by nanoparticle growth.^24^ This two-stage evolution—rapid nucleation followed by slower growth—suggests a sequential formation process under ambient conditions. The SPR maximum remains nearly constant (≈530–538 nm) with only a slight redshift over the course of the reaction, consistent with moderate particle growth and limited interparticle coupling. The stability of the plasmon maximum indicates stable particle formation without extensive aggregation.^25^

In Fig. 2B, plotting the absorbance intensity at ∼530 nm as a function of time reveals a clear time-dependent correlation between reaction progression and plasmonic development. Together, these observations highlight the capability of SiNPs@2-propynylamine to mediate AuNP growth without the need for external reductants, demonstrating both the chemical activity of the surface ligands and the dynamic evolution of plasmonic properties.^7–9^

These spectral variations, along with the visible color evolution of the reaction mixture shown in Fig. 2C, qualitatively confirm a stepwise nanoparticle formation process. The gradual increase in absorbance intensity and the narrowing of the plasmon band indicate a continuous nucleation and growth process during the first hour of reaction.

**Fig. 3:**
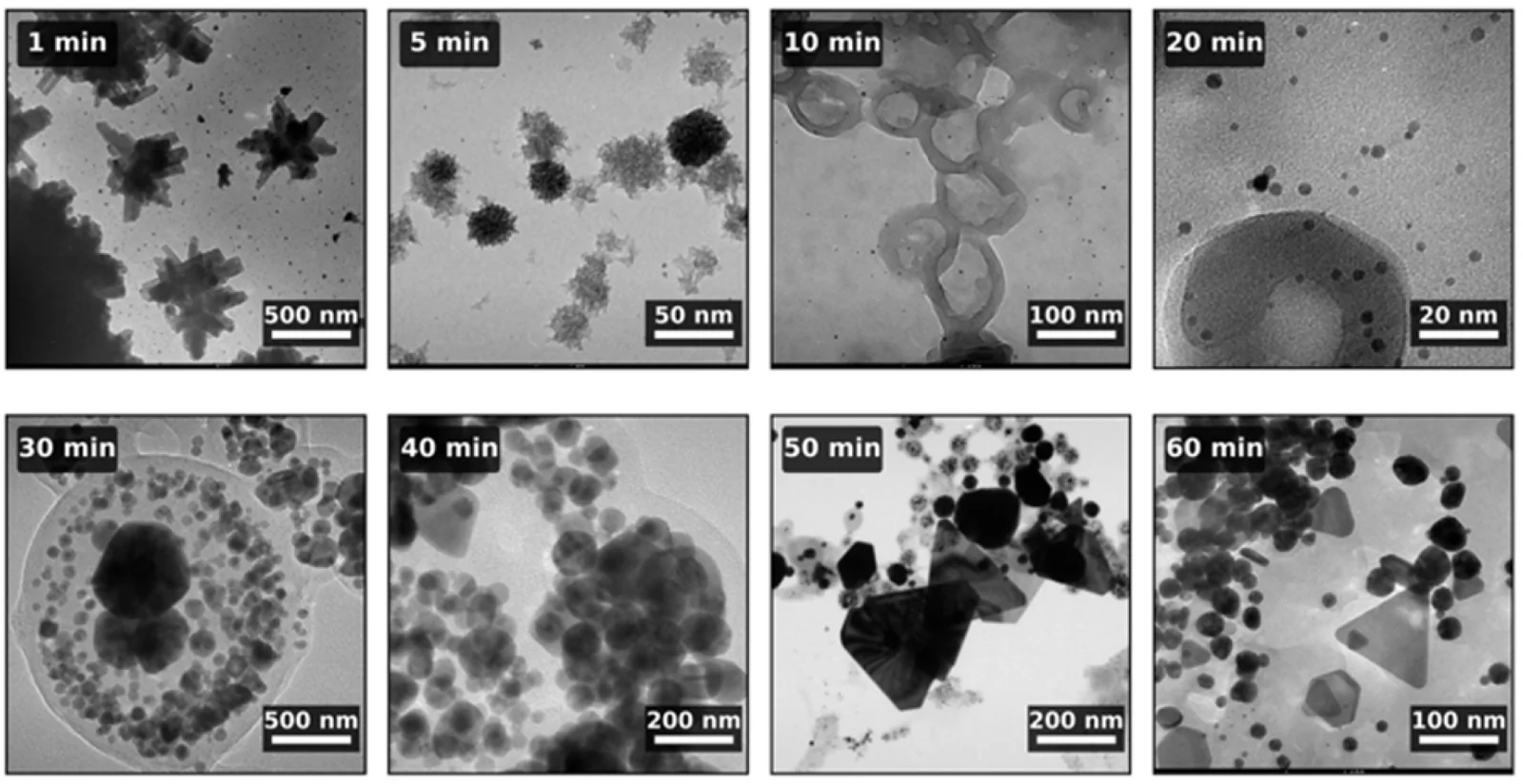
Morphological evolution of AuNPs synthesized *via* the reduction of HAuCl_4_·3H_2_O by SiNPs@2-propynylamine at 20 °C. Representative TEM images collected at 1, 5, 10, 20, 30, 40, 50, and 60 min illustrate the progressive growth, aggregation, and reorganization behaviors of AuNPs on the SiNP surface. Scale is as indicated in each panel.

TEM images acquired between 1 and 60 min, shown in Fig. 3, reveal a clear sequential evolution in both the morphology and size of the synthesized AuNPs. At 1 min, small nuclei are only faintly discernible as scattered dark contrast features. By 5 min, dendritic and branched aggregates dominate the field of view, consistent with rapid, non-equilibrium nucleation under seed-free conditions.^26,27^ Between 10 and 30 min, hollow and shell-like structures become prevalent, accompanied by large heterogeneous domains exhibiting internal texture and progressive fragmentation. By 40 min, the population transitions into more compact aggregates, with the morphology evolving toward well-defined faceted particles (including triangular and polyhedral geometries), with smaller near-spherical nanoparticles distributed throughout the field of view by the end of the experiment period (50–60 min). This temporal progression, which advances from nuclei to dendritic aggregates, hollow shells, compact structures, and ultimately to faceted and spherical nanoparticles, reflects the gradual shift of the system toward thermodynamically favored morphologies as the concentration of Au^3+^ precursors decreases.

Quantitative analysis of 652 particles across 12 representative micrographs (Fig. S6–S9 and Table S5) confirms that the final population is dominated by spherical (43.4%) and near-spherical (30.5%) nanoparticles, with faceted polyhedral particles representing 3.8% and dendritic, hollow, and rod-like morphologies together accounting for ∼2.4%; irregular aggregates contribute the remaining 19.8%. As the reaction progresses (30 to 50 min), these large assemblies undergo significant reorganization and partial disintegration. This change in architecture is evidenced by the emergence of smaller, more uniform particles, often templated or confined by the SiNP supports. This refinement in particle size reflects the natural progression of nanoparticle evolution and aligns with the reorganization processes frequently observed during nanomaterial growth.^23,24^ The available data clearly capture this transition, though assigning a specific pathway is beyond the scope of the present study.^28,29^

Although our TEM analysis reveals a diversity of AuNP morphologies, the UV-vis spectra display only a single SPR band near 530 nm. Despite the moderate anisotropy and deviations from perfect sphericity present in some structures, it is likely that these morphologies do not reach the level of shape elongation or sharpness required to produce additional, well-resolved plasmon bands.^31^ Instead, the anisotropy present in these structures broadens or slightly shifts the principal SPR band without generating separate peaks.^32^ This behavior is well documented for compact or moderately polyhedral AuNPs such as cubes, concave cubes, hollow structures, and polygonal forms, where a single plasmon band dominates the optical response.^33^ Similarly to the UV-vis results, TEM results suggest a mechanistic pathway involving rapid nucleation and aggregation, followed by surface-driven reorganization and size homogenization, leading to stable AuNPs with well-defined SPR features.^30^

DLS provided complementary, ensemble-level size information for the AuNPs@SiNPs@2-propynylamine hybrid. The intensity-weighted hydrodynamic diameter (*D*_h_) was 165 ± 12 nm with a polydispersity index of 24.5%, consistent with a weakly bimodal distribution in which a minor sub-population near 10 nm (individual SiNPs/AuNPs) coexists with a dominant population of a few hundred nanometers (hybrid assemblies).^23^ These ensemble values are substantially larger than the primary particle sizes resolved by TEM (mean 3.7 ± 1.1 nm for SiNPs, Fig. S3), as expected for an intensity-weighted technique that is strongly biased toward larger scatterers.^20–23^ The DLS data therefore qualitatively corroborate the broad size distribution and partial aggregation observed by TEM. Full DLS distributions and colloidal parameters are presented in Fig. S10, S11 and Table S6 of the SI.

### Spatial and compositional analysis of Au–Si nanostructures

**Fig. 4:**
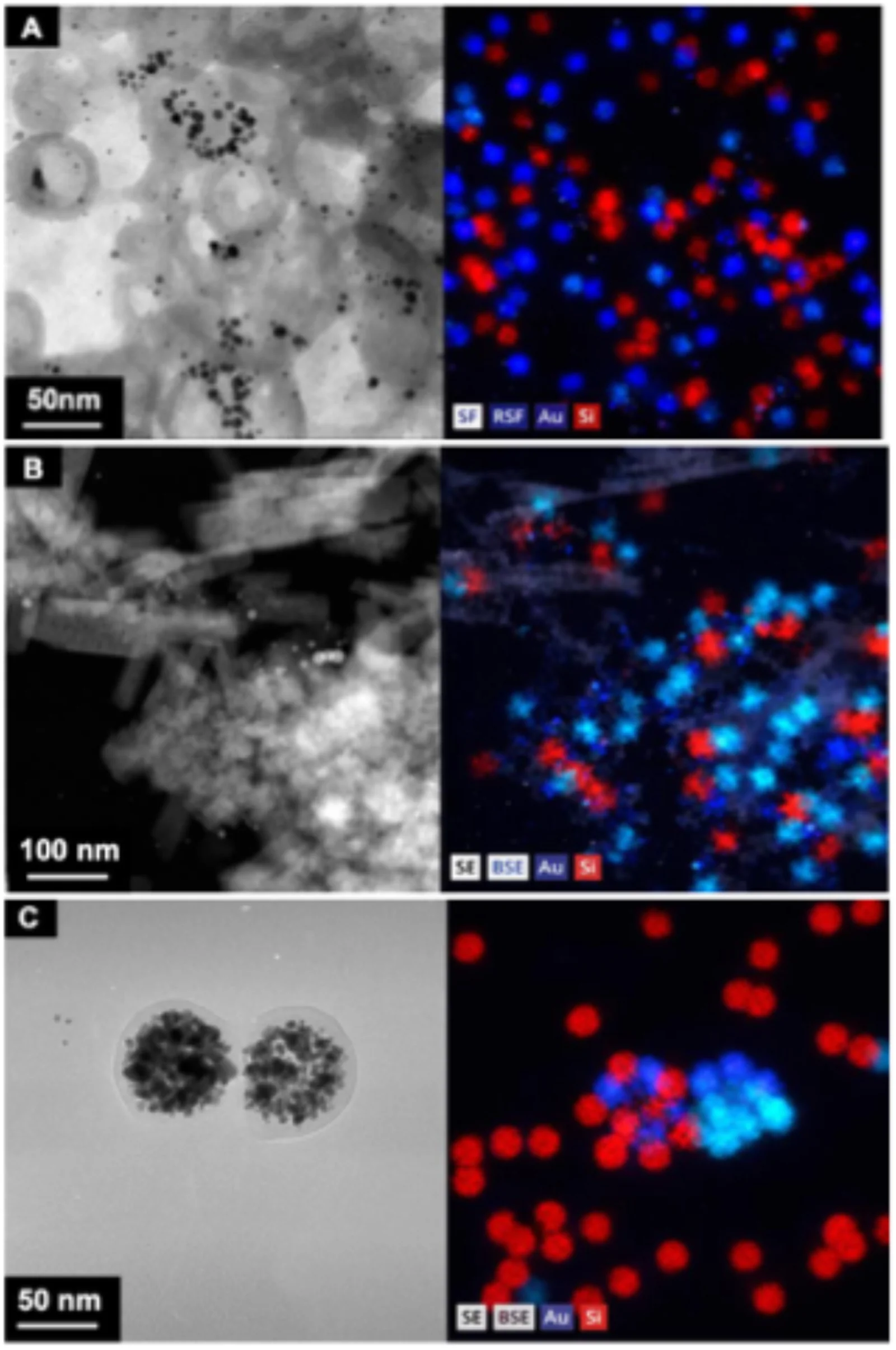
EDS elemental mapping and corresponding TEM images of Au–Si nanostructures synthesized with SiNPs@2-propynylamine. Elemental maps (left) and TEM images (right) show the progressive enrichment of Au (blue) on Si-rich domains (red). Early aggregates (A and B) display partial Au deposition, while later structures (C) reveal faceted AuNPs with minimal Si signal, consistent with complete reduction and higher electron scattering of Au.

EDS mapping of the Au–Si nanostructures shown in Fig. 4 confirms the morphological evolution observed by TEM, validating the compositional progression from Si-assisted nucleation to Au domain growth. In the early stages (Fig. 4A and B), the Si signal appears as discrete maxima distributed across the mapped area, spatially correlated with the heterogeneous dendritic Au aggregates observed by TEM (1–20 min). Given the limited spatial resolution of EDS and the comparatively large X-ray interaction volume, these Si features cannot be unambiguously assigned to individual ∼10 nm SiNPs, but are nevertheless consistent with nucleation initiating at SiNP-rich domains rather than through homogeneous solution processes.^24^

At the later stages (Fig. 4C), Au increasingly overlaps the Si regions, consistent with the restructuring and densification observed in TEM (30–50 min). This partial coverage suggests progressive surface growth and coalescence of Au domains, leading to the formation of compact, faceted nanoparticles. Within these same later-stage maps (Fig. 4C), Au dominates the EDS maps as Si signals weaken. This effect is attributed to the stronger electron scattering and X-ray attenuation of gold rather than the absence of silicon.^34^ The EDS results confirm a surface-mediated mechanism in which SiNPs facilitate localized Au reduction and subsequent growth into independent, well-faceted nanoparticles, fully aligning with the sequential morphological evolution revealed by TEM.

### FTIR evidence of coordination and redox pathways

**Fig. 5:**
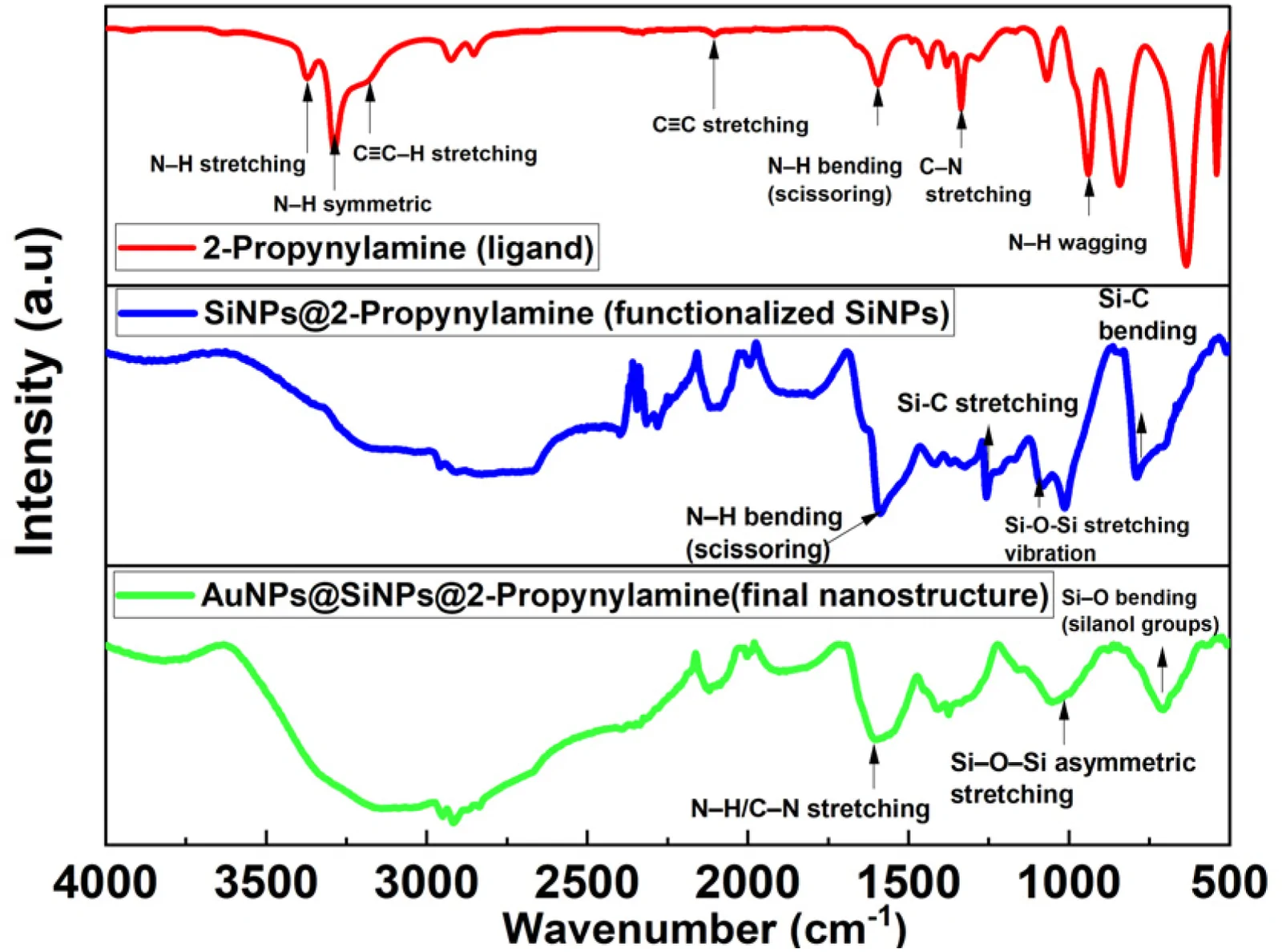
ATR-FTIR spectra of free 2-propynylamine (reference sample), SiNPs@2-propynylamine (after 1 h of ligand grafting), and AuNPs@SiNPs@2-propynylamine hybrid (after 1 h of Au deposition). Spectra were collected in ATR mode and plotted as negative intensity peaks.

ATR-FTIR spectra were collected after 1 hour of reaction and are displayed in Fig. 5 as negative intensity peaks. The spectrum of free 2-propynylamine exhibits all the characteristic vibrations of a terminal-alkyne-bearing primary amine, including N–H stretching bands at 3368 and 3281 cm^−1^ and a prominent C≡C stretching band at 2104 cm^−1^, confirming the presence of the terminal alkyne group. Additional absorptions, such as CH_2_ bending (1437 cm^−1^), C–N stretching (1064 cm^−1^), and N–H wagging (940 cm^−1^), match reported data for terminal alkynylamines.^35^

The spectral profile for SiNPs@2-propynylamine shows distinct changes indicative of surface modification. The disappearance of the diagnostic C≡C stretching band (2104 cm^−1^) indicates chemical bonding or reaction of the alkyne group with surface silicon atoms. In addition, characteristic Si–C vibrational features appear at ∼1251 cm^−1^ (Si–C stretching, partially overlapped with Si–O modes) and ∼790 cm^−1^ (Si–C bending), consistent with covalent attachment of the ligand to the silicon surface.^36,37^ The N–H stretching envelope shifts slightly to lower wavenumbers (3345–3275 cm^−1^), suggesting hydrogen bonding between the amine group and oxidized Si–OH sites.^38^ The N–H bending mode shifts from 1591 to 1544 cm^−1^, supporting amine–surface interaction.^39^ The Si–C bending (∼790 cm^−1^) and Si–O bands (∼1040 cm^−1^) remain visible, indicating preservation of the silicon core and increased surface oxidation during AuNP deposition.^40,41^

The FTIR spectrum of the hybrid AuNPs@SiNPs@2-propynylamine reveals additional changes attributable to amine–gold interactions after AuNP formation. The N–H stretching region becomes broader and less intense (3330–3260 cm^−1^), while the N–H bending vibration shifts to 1604 cm^−1^. A concurrent shift of the C–N stretching band from 1087 to 1047 cm^−1^ reflects a redistribution of electron density upon coordination to gold.^35,37^ These features, absent in the SiNPs@2-propynylamine spectrum, indicate that the grafted ligand participates in stabilizing the resulting Au–Si hybrid structure. Vibrational assignments are summarized in Table S7 of the SI.

### XPS analysis

**Fig. 6:**
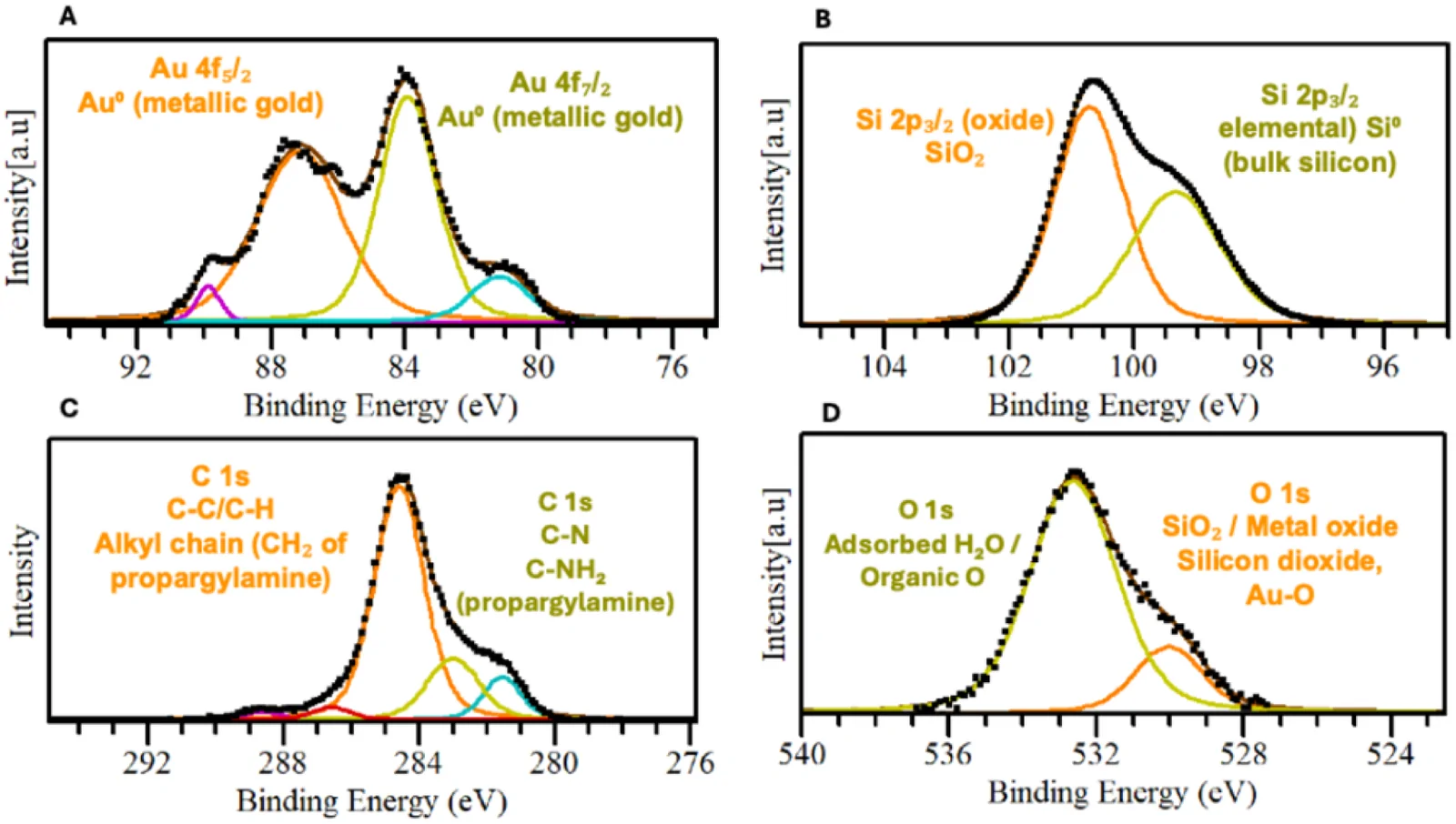
XPS spectra of AuNPs@SiNPs@2-propynylamine. (A) Au 4f region showing metallic Au^0^ and a higher-binding-energy component attributed to surface-oxidized gold species. (B) Si 2p region resolved into Si^0^ and suboxide components (Si^+^–Si^3+^) together with a higher-binding-energy contribution arising from partial surface oxidation. (C) C 1s region deconvoluted into C–C/C–H and Si–C environments. (D) O 1s region displaying signals associated with Si–O and Si–OH species typical of partially oxidized silicon surfaces.

XPS analysis provides direct insight into the electronic and interfacial structure of the hybrid AuNPs@SiNPs@2-propynylamine system (Fig. 6), corroborating the FTIR findings. The XPS data reveal the coexistence of metallic Au^0^ and higher-binding-energy oxidized gold species, indicative of partial surface oxidation during nanoparticle growth.^42^ Quantitative peak fitting results are provided in Tables S8–S11 of the SI.

The Au 4f spectrum (Fig. 6A) displays two doublets corresponding to metallic and partially oxidized gold species. The main doublet at 84.3 eV (4f_7/2_) and 87.8 eV (4f_5/2_), representing ∼47% of the integrated area, is assigned to metallic Au^0^.^43,44^ The second, higher-energy doublet (∼86.4 and 90.1 eV), accounting for ∼12% of the area, is attributed to surface-oxidized Au species commonly observed in ligand-containing or aqueous environments, while the remaining intensity is associated with inelastic scattering contributions and unresolved interfacial gold states. The coexistence of these components suggests that the reduction of Au^3+^ is not complete under the reaction conditions. These XPS results are compatible with the TEM observations and with the evolution of the UV-vis spectra, indicating that the gold surface remains chemically active during the early stages of nanoparticle growth.^45^ These findings also align with the TEM observations and the gradual stabilization of SPR in UV-vis spectra, further substantiating the dynamic surface chemistry during synthesis.

Analysis of the Si 2p region (Fig. 6B) reveals three prominent components, suggesting a partially oxidized silicon surface. A low binding-energy peak at ∼99.3 eV (∼30%) corresponds to Si^0^ and reflects the silicon-rich core. The component at ∼100–101 eV (∼67%) is attributed to suboxide species (Si^+^–Si^3+^), which normally arise from partial surface oxidation. The highest-energy contribution, ∼103.5 eV (∼3%), indicates additional oxidized environments but remains compatible with a thin, non-stoichiometric surface oxide layer rather than fully developed SiO_2_.^46–48^ This distribution is typical for functionalized SiNPs and supports the presence of Si–C bonds, as well as minor surface oxidation, consistent with the weak intensity of the Si–O stretching band in the FTIR spectra. These results confirm that the SiNPs possess a predominantly Si–C-terminated surface with localized regions of suboxide formed upon exposure to ambient oxygen.^49^ The observation of multiple oxidation states is corroborated by the partial surface oxidation of silicon prior to and during gold deposition, in agreement with the EDS mapping.

In the C 1s spectrum of the functionalized SiNPs (Fig. 6C), the main peak at ∼284.9 eV is assigned to C–C and C–H environments originating from adventitious carbon and organic surface species.^48,50^ A second component near 282.0 eV is characteristic of Si–C bonds and is consistent with covalent attachment of the 2-propynylamine ligand to the silicon surface.^51^ A weaker contribution around 285 eV corresponds to additional C–C/C–H environments commonly observed on nanoparticle surfaces.^52^ The presence of the low-binding-energy Si–C feature provides direct evidence of covalent Si–C bond formation and confirms effective chemical modification of the silicon surface.

The O 1s spectrum (Fig. 6D) exhibits two components characteristic of partially oxidized silicon surfaces. The main peak at ∼532.8 eV is assigned to Si–O and Si–OH species, which commonly arise from native oxidation and exposure of the functionalized SiNPs to aqueous media.^53^ A second, lower-binding-energy component near ∼531.2 eV is attributed to additional oxygen environments associated with non-stoichiometric suboxide regions.^54,55^

These XPS results indicate the coexistence of metallic Au^0^ and higher-binding-energy oxidized gold species. Notably, the latter are shifted to higher binding energies, consistent with partial surface oxidation and ligand–metal interactions. These findings, along with the presence of silicon in multiple oxidation states, are consistent with a partially oxidized silicon surface that supports the deposited gold domains. The presence of Si–C bonds at ∼282 eV confirms chemical linkage between the SiNPs and the 2-propynylamine. Partial surface oxidation and covalent interactions define the composition and connectivity at the Au–Si interface. Detailed peak positions, full width at half maximum relative areas, and corresponding assignments for gold, silicon, carbon, and oxygen are summarized in Tables S8–S11 of the SI; survey XPS spectra of all characterized materials are provided in Fig. S12. The progressive evolution of the elemental composition across the sample series (from oxide-terminated silicon precursors, through nitrogen-bearing SiNPs@2-propynylamine, to the gold- and nitrogen-containing hybrid) provides survey-level corroboration of the stepwise surface functionalization and interfacial gold deposition described herein.

### Proposed interfacial redox mechanism for AuNP formation mediated by SiNPs@2-propynylamine

**Fig. 7:**
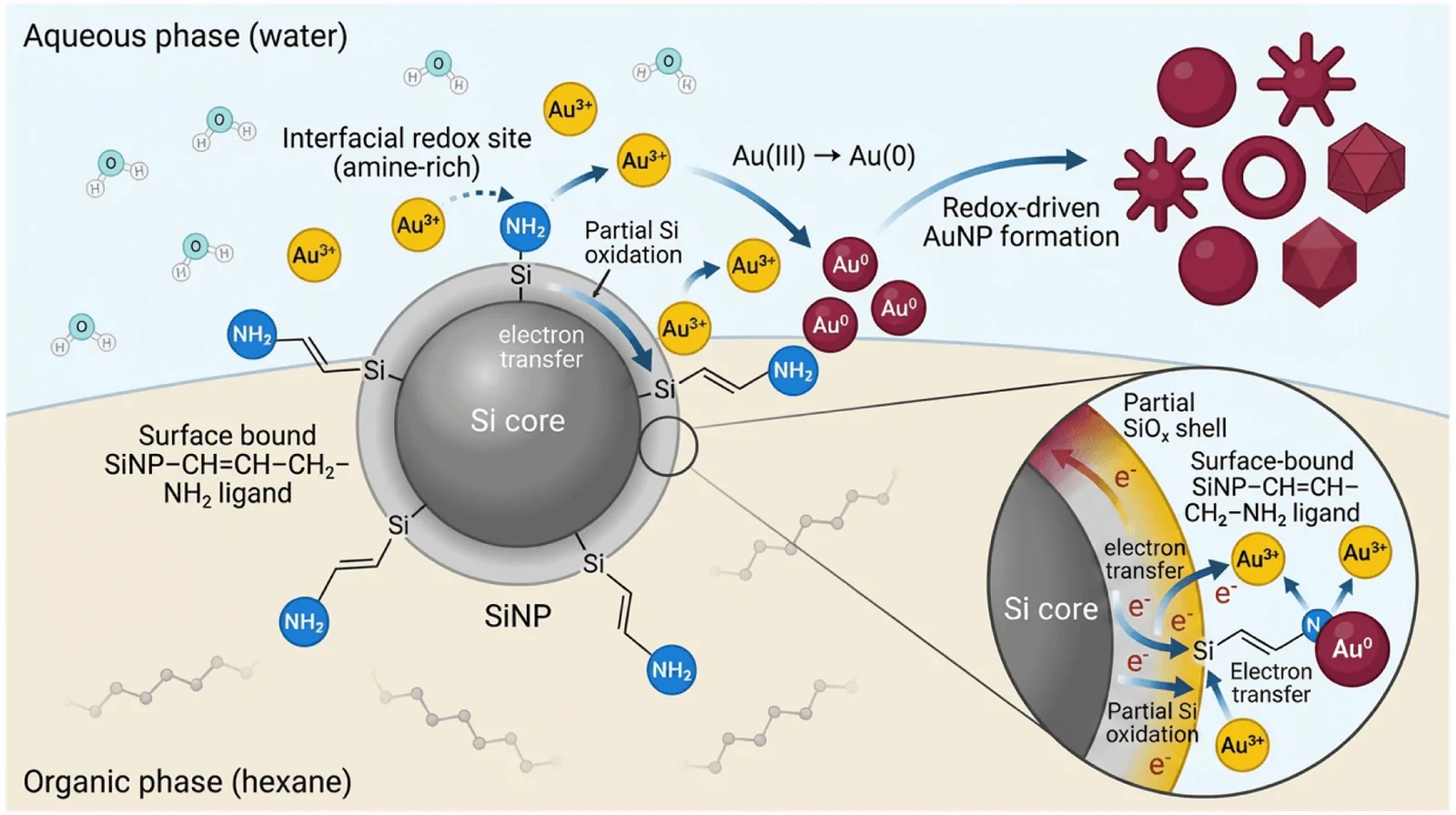
Proposed mechanism for Au–Si hybrid nanostructure formation *via* AuNP growth on functionalized SiNPs.

The combined spectroscopic and microscopic data confirm that the formation of AuNPs@SiNPs@2-propynylamine follows an interfacial redox mechanism driven by the partially oxidized silicon surface (Fig. 7).^11,18,19^ The alkyne group of 2-propynylamine anchored to the Si surface atoms through a Si–C linkage, as previously established, enables electron transfer between the SiNP and aqueous gold.^40,48^ UV-vis monitoring during synthesis displays the gradual emergence and stabilization of an SPR band between 530–550 nm, corresponding to the nucleation and growth of metallic Au domains.^21,22,29^ TEM and EDS analyses reveal that these domains evolve from large, irregular aggregates into smaller, faceted nanoparticles, suggesting that gold is transiently reduced at the oxidized SiNP surface and subsequently released into solution rather than progressively deposited on the silicon oxide.^27,41^ Lastly, XPS provides direct evidence of the redox coupling that drives this process; the coexistence of Au^0^ and Au^3+^ species, together with Si^0^ and suboxide states (Si^+^–Si^3+^) confirms that electrons originating from partially reduced silicon are transferred to Au^3+^ ions during nanoparticle formation.^44,47,55^ This correlated spectroscopic and structural evidence establishes that Au growth is governed by a surface-mediated charge-transfer process in which silicon oxidation and gold reduction occur simultaneously at the solid–liquid interface. In a biphasic hexane–water environment, coordination of Au^3+^ is expected to occur predominantly at the amine-terminated ligand sites exposed to the aqueous phase, rather than uniformly across the entire nanoparticle surface.

At the initial stage of AuNP growth on SiNPs@2-propynylamine, TEM images reveal large dendritic and hollow aggregates, characteristic of rapid nucleation and uncontrolled aggregation. FTIR and XPS analyses confirm covalent binding of 2-propynylamine to the silicon surface and the formation of a mostly oxidized silicon shell. This bonding environment enables interfacial electron transfer leading to the reduction of Au precursors. In addition, an SPR at ∼530 nm in the UV-vis spectrum signals the emergence of metallic gold domains within the hybrid structure.

At the intermediate stage of the reaction, TEM and EDS illustrate that the large aggregates disintegrate into smaller, more uniformly distributed gold domains on SiNPs@2-propynylamine. EDS mapping reveals an increased gold signal and attenuated silicon contribution, in line with progressive gold deposition on oxidized silicon surfaces.^12,24,42^ FTIR spectra display a slight broadening of bands in the 3345–3260 cm^−1^ region, indicating enhanced hydrogen bonding and surface interactions. XPS detects the coexistence of Au^0^ and Au^3+^ species, consistent with ongoing interfacial electron transfer as the silicon surface becomes further oxidized.^17,42,55^ The UV-vis spectra show a gradual increase in plasmon intensity with a nearly constant band maximum at ∼530–538 nm, reflecting sequential nucleation and moderate growth of AuNPs on the SiNP surface without pronounced interparticle coupling or aggregation.

The TEM images indicate that the final product contains predominantly spherical and faceted AuNPs, consistent with a transition to stable, well-defined morphologies. EDS shows a strong gold signal and attenuated silicon intensity, consistent with the advanced stages of gold domain growth and the high electron-scattering contrast of Au relative to Si.^11,41^ XPS indicates progression toward higher oxidation states but remains consistent with non-stoichiometric SiO_*x*_, rather than fully developed SiO_2_. Gold remains partly oxidized, with metallic Au^0^ and higher-binding-energy oxidized species present, evidencing a persistent interfacial equilibrium between metallic and oxidized species.^42,46^ Finally, the UV-vis SPR band stabilizes near 530 nm, consistent with the sequential growth of AuNPs and the optical response of well-dispersed metallic nanoparticles. Collectively, these results confirm that gold reduction and silicon oxidation occur simultaneously through interfacial charge transfer, resulting in the formation of stable Au–Si hybrid nanostructures.^10–12^

## Conclusions

This work establishes amine-functionalized SiNPs as integrated redox mediators and transient nucleation platforms for the surfactant-free, time-dependent formation of gold nanoparticles under ambient conditions. The combined spectroscopic and microscopic analyses demonstrate that electron transfer from partially oxidized silicon surfaces drives the stepwise reduction of Au^3+^ to Au^0^, giving rise to a temporal morphological evolution in which the final nanoparticle population is dominated by spherical and near-spherical particles, with faceted polyhedral structures persisting as a minor but visually prominent fraction. This interfacial redox process, which exploits the intrinsic surface oxidation of silicon in aqueous environments and the presence of covalently grafted primary amine ligands, provides a sustainable, ligand-assisted pathway to Au–Si hybrid nanostructures. More broadly, this strategy offers a versatile platform for engineering tunable charge-transfer interfaces with potential impacts in plasmonic sensing, heterogeneous catalysis, and photonic or optoelectronic device architectures.

## Author contributions

A. G. synthesized the precursor and AuNPs@SiNPs@2-propynylamine and characterized the oxidation of the surfaces by XPS. B. G. carried out the control experiments and analysis with FTIR and UV-vis. B. M. and M. F. provided valuable advice on the characterization techniques, specifically regarding XPS. J. V. designed the research plan, supervised the research, and contributed to writing and editing the manuscript. J. V. also conducted TEM and EDS analyses and data processing. All authors reviewed and approved the manuscript.

## Conflicts of interest

There are no conflicts to declare.

## Supplementary Material

NA-008-D6NA00512H-s001

## Data Availability

The data supporting this article have been included as part of the supplementary information (SI). All raw and processed data, including UV-vis spectra, TEM images, DLS measurements, XPS spectra, FTIR spectra, and morphological analysis files, are available in the SI accompanying this manuscript. Additional data related to this study may be obtained from the corresponding author upon reasonable request. Supplementary information: expeimental procedures, control experiments, UV-vis, TEM, DLS, FTIR and XPS characterization, quantitative morphology analysis, XPS peak fitting and oxide-thickness calculations, supporting tables and additiona discussion supporting the conclusions of this work. See DOI: https://doi.org/10.1039/d6na00512h.
