# Supplementary material for: Interfacial redox mediation by amine/alkyne-functionalized silicon nanoparticles: surfactant-free gold nanoparticle synthesis
Source: Nanoscale Adv. 2026 Jul 1. Online ahead of print. doi: 10.1039/d6na00512h (PMC13338794; doi:10.1039/d6na00512h)
Supplement: NA-OLF-D6NA00512H-s001 [file NA-OLF-D6NA00512H-s001.pdf]

Supplementary Information (SI) for Nanoscale Advances.
This journal is © The Royal Society of Chemistry 2026

Supplementary Materials for

Interfacial Redox Mediation by Amine/Alkyne-Functionalized Silicon Nanoparticles: Surfactant-Free Gold Nanoparticle Synthesis

Amber L. Garcia[1], Brittany Griggs[2], Brian S. Mitchell[3], Mark J. Fink[4], and Julie P. Vanegas[5]*

1. College of Engineering and Computer Science, The University of Texas Rio Grande Valley, Edinburg, TX 78539
2. School of Integrative Biological and Chemical Sciences, The University of Texas Rio Grande Valley, Edinburg, TX 78539
3. Department of Chemical and Biomolecular Engineering, Tulane University, New Orleans, LA 70118
4. Department of Chemistry, Tulane University, New Orleans, LA 70118
5. Department of Physics and Astronomy, The University of Texas Rio Grande Valley, Edinburg, TX 78539

*Corresponding Author

Julie P. Vanegas. University of Texas Rio Grande Valley. Department of Physics and Astronomy. University of Texas Rio Grande Valley. Science Building. 1201 W University Dr, Edinburg, TX 78539 (USA) E-mail: Julie.vanegas@utrgv.edu ORCID #0000-0002-5811-1382

Synthesis and Characterization of SiNPs Passivated with 2-Propynylamine

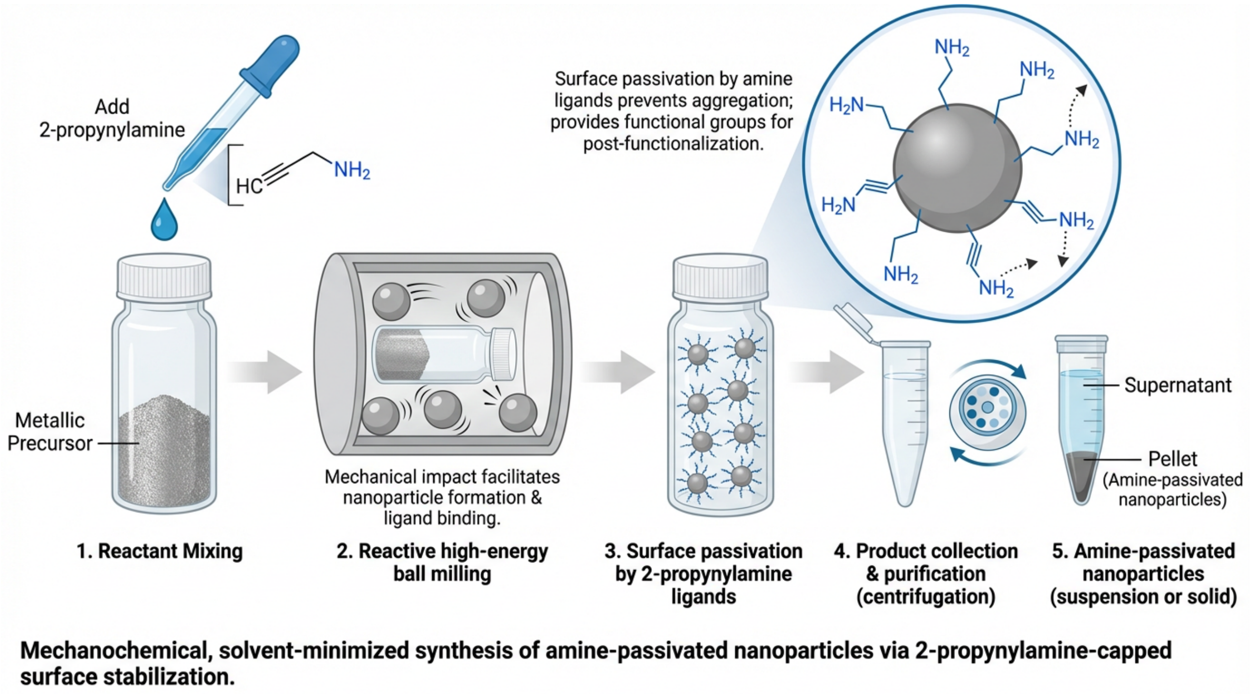

Figure S1. *Illustration of the solvent-minimized mechanochemical approach for synthesizing amine-passivated silicon nanoparticles. The process involves (i) mixing of the metallic precursor with 2-propynylamine, (ii) reactive high-energy ball milling to drive nanoparticle formation and ligand attachment, (iii) surface passivation by amine groups to prevent aggregation, (iv) centrifugation-based purification, and (v) isolation of the functionalized SiNPs.*

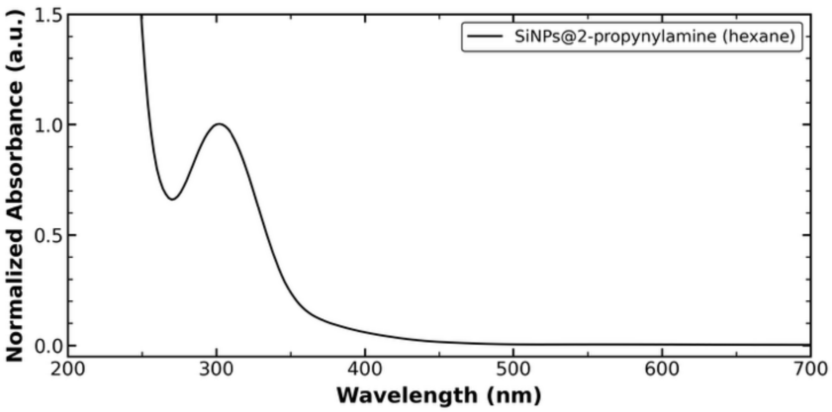

Figure S2. *UV-Vis spectrum confirming the optical properties of 2-propynylamine-capped SiNPs dispersed in hexane.*

Figure S3. *TEM micrograph and size distribution histogram of SiNPs@2-propynylamine. Inset: particle size analysis showing mean diameter of 3.7 ± 1.1 nm and median of 3.0 nm.*

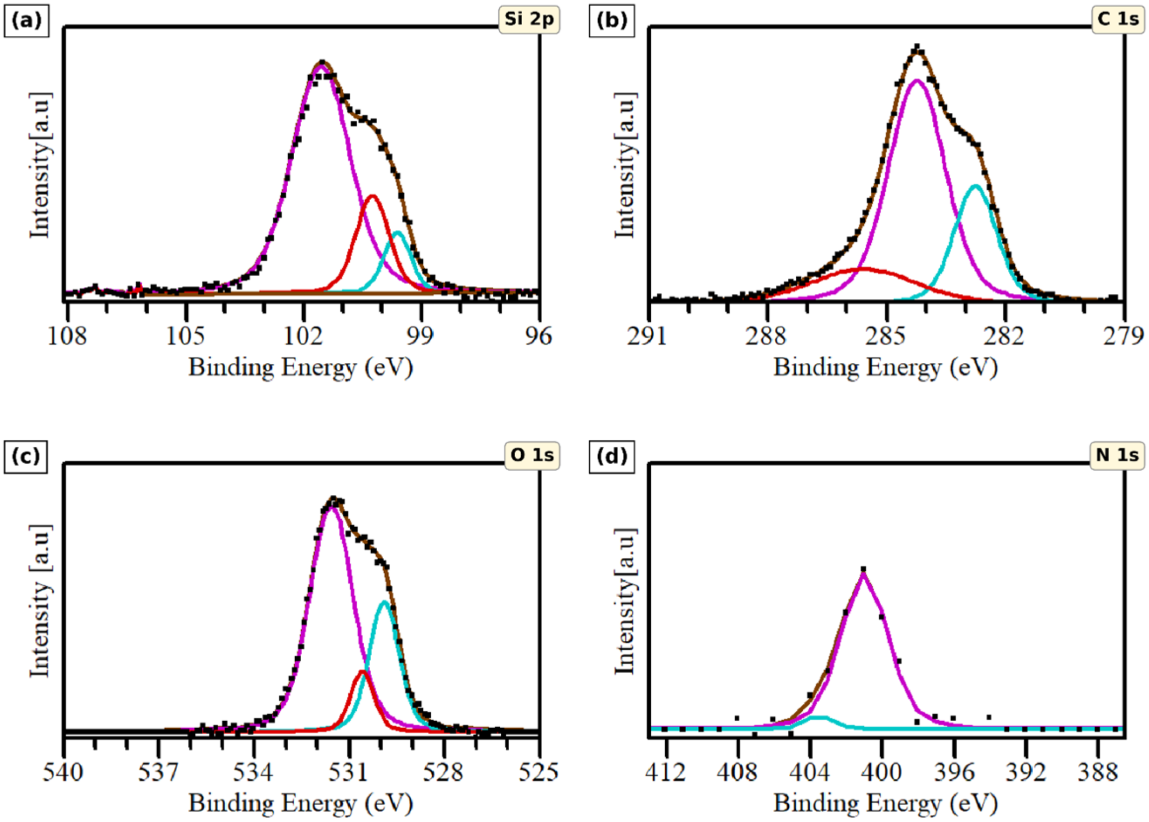

Figure S4. *Deconvoluted high-resolution XPS spectra of SiNPs@2-propynylamine. (a) Si 2p core-level spectrum deconvoluted into three Si environments at 99.62 eV (Si–C/Si^0), 100.25 eV (Si^+/Si^{2+} suboxides), and 101.56 eV (Si^{3+}). (b) C 1s spectrum showing contributions from C–C/C–H, C–N, and C–O bonding. (c) O 1s spectrum revealing Si–O–Si and surface hydroxyl species. (d) N 1s spectrum confirming the presence of amine (–NH_2) groups from 2-propynylamine functionalization.*

Table S1. High-resolution XPS peak fitting parameters for SiNPs@2-propynylamine.

Core Level	Binding Energy (eV)	Assignment	Area (%)
**Si 2p**	99.62	Si–C / Si^0	9.10
**Si 2p**	100.25	Si^+/Si^{2+} (suboxides)	17.96
**Si 2p**	101.56	Si^{3+} (higher suboxides)	72.94
**C 1s**	284.8	C–C / C–H	45.2
**C 1s**	286.2	C–N / C–O	35.8
**C 1s**	288.5	O–C=O / π–π*	19.0
**O 1s**	531.2	Si–O–Si	55.3
**O 1s**	532.5	Si–O–H / C=O	30.1
**O 1s**	533.8	H_2O / adsorbed O	14.6
**N 1s**	399.5	$–NH_2$ (amine)	78.5
**N 1s**	401.2	$–NH_3^+$ / protonated amine	21.5

Calculation of the native-oxide thickness

The thickness of the SiO_2 layer was estimated from the Si 2p high-resolution spectra using the standard model proposed by Hill et al.[1,2] and applied to native silicon oxide by Strohmeier[3] (1990):

$$d_{SiO_2} = L_{eff} \cdot \sin\theta \cdot \ln[\, 1 + I_{Si^{4+}} / I_{Si^0} \,]$$

where L_{eff} is the effective inelastic mean free path (IMFP) of Si 2p photoelectrons through SiO_2, θ is the photoelectron take-off angle and $I_{Si^{4+}}$ / I_{Si^0} is the integrated-area ratio of the oxidized and elemental Si components obtained from the deconvolution of the Si 2p spectrum. For Al Kα radiation (1486.6 eV) and normal emission (θ = 90°), an effective value of L_{eff} = 2.6 nm has been adopted; this value already incorporates the IMFPs of Si 2p photoelectrons in both SiO_2 (~3.4 nm) and Si (~2.96 nm) and the corresponding atomic-density correction factor. For the etched wafer, in which a sub-oxide component (Si^{+}–Si^{3+}) is also resolved at ≈101.9 eV, only the dominant fully oxidized Si^{4+} and elemental Si^0 components were used for the thickness calculation; the sub-oxide is reported separately in Table S8.

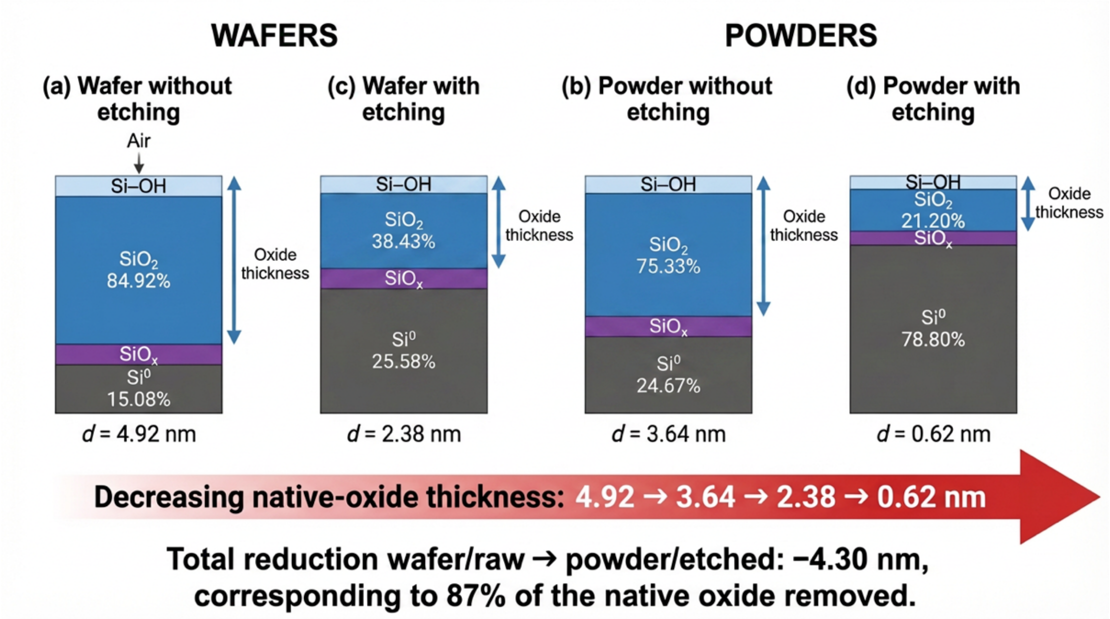

Figure S5. *XPS-derived surface layer model of silicon wafers and powders before and after etching. Four precursor states are compared, showing the progressive decrease in native SiO_2 thickness estimated from Si 2p. (a) The unetched wafer shows the thickest oxide (d = 4.92 nm). (b) The unetched powder exhibits a reduced layer (d = 3.64 nm), indicating partial oxide removal from powder processing. (c) After chemical etching, the wafer oxide decreases to d = 2.38 nm, with an interfacial SiO_x contribution becoming evident. (d) The etched powder shows the greatest removal (d = 0.62 nm), with Si^0 dominating the Si 2p signal (78.80%). Overall, oxide thickness decreases from 4.92 to 0.62 nm (~87%). The SiO_x layer is included as an interfacial region between SiO_2 and Si^0 and is directly resolved by XPS only in the etched wafer.*

Substituting the integrated areas obtained from the Si 2p deconvolution (Figure S5 Table S2) into the Hill–Strohmeier equation yields[3] d_{SiO_2} = 4.92 nm for the unetched wafer, 3.64 nm for the unetched powder, 2.38 nm for the etched wafer and 0.62 nm for the etched powder. The trend is monotonic and chemically consistent: chemical etching reduces the native oxide layer in both forms, but the effect is much stronger for the powder due to its higher surface-to-volume ratio and the improved accessibility of the etching solution to the oxide. The etched powder, which is the precursor entering the RHEBM step, retains only ≈0.6 nm of residual oxide — corresponding to an 87 % reduction relative to the as-received wafer. This near-bare Si^0 surface is the chemical starting point that is subsequently consumed and reorganized during RHEBM with 2-propynylamine: in the final SiNPs@2-propynylamine product the fully oxidized Si^{4+} component (~103 eV) is no longer detectable, and the Si 2p spectrum is dominated by Si–C / Si^0 (9.10 %), low-valent suboxides Si^{+}/Si^{2+} (17.96 %) and Si^{3+} (72.94 %) environments (main-text Figure 6, Table S1).

XPS peak-fitting parameters of the silicon precursors

Table S2 lists the binding energy (BE), integrated peak area, % area within each region, and chemical assignment for every component obtained from the deconvolution of the Si 2p, O 1s and C 1s high-resolution spectra of the four samples. Mixed Gaussian–Lorentzian peak shapes were used throughout, in agreement with the procedure described in the main-text Methods section.

Table S2. XPS fitting parameters for the four silicon precursor states.

Sample	Region	BE (eV)	Area	% Area	Assignment
**Wafer without etching**	Si 2p	102.98	9610.7	84.92	Si^{4+} (SiO_2, native oxide)
**Wafer without etching**	Si 2p	98.89	1707.2	15.08	Si^0 (crystalline substrate)
**Wafer without etching**	O 1s	531.92	48809.7	63.52	Si–O–Si (silica network)
**Wafer without etching**	O 1s	532.58	28036.0	36.48	Si–OH (terminal silanols)
**Wafer without etching**	C 1s	284.59	12260.7	57.86	C–C / C–H
**Wafer without etching**	C 1s	285.86	8929.4	42.14	C–O / adventitious
**Wafer with etching**	Si 2p	102.91	2597.6	38.41	Si^{4+} (residual SiO_2)
**Wafer with etching**	Si 2p	101.89	2434.8	36.01	Si^{+}–Si^{3+} sub-oxide
**Wafer with etching**	Si 2p	98.76	1729.6	25.58	Si^0
**Wafer with etching**	O 1s	531.05	27458.1	42.46	Si–O / sub-oxide O
**Wafer with etching**	O 1s	532.66	37207.4	57.54	Si–OH / Si–O–Si
**Wafer with etching**	C 1s	284.73	18080.5	73.04	C–C / C–H
**Wafer with etching**	C 1s	286.10	6674.1	26.96	C–O / adventitious
**Powder without etching**	Si 2p	102.49	5505.9	75.33	Si^{4+} (oxidised powder)
**Powder without etching**	Si 2p	98.74	1803.4	24.67	Si^0
**Powder without etching**	O 1s	532.13	45485.5	69.56	Si–O–Si
**Powder without etching**	O 1s	533.14	8842.6	13.52	Si–OH
**Powder without etching**	O 1s	530.62	11059.4	16.91	Surface–OH / loosely-bound O
**Powder without etching**	C 1s	284.68	12735.2	47.63	C–C / C–H
**Powder without etching**	C 1s	285.61	14004.6	52.37	C–O / adventitious
**Powder with etching**	Si 2p	102.59	2983.0	21.20	Si^{4+} (thin residual oxide)

**Powder with etching**	Si 2p	98.67	11090.9	78.80	Si^0 (dominant)
**Powder with etching**	O 1s	532.83	12814.0	39.86	Si–OH / Si–O–Si
**Powder with etching**	O 1s	531.62	19332.6	60.14	Si–O / sub-oxide O
**Powder with etching**	C 1s	284.21	3328.2	44.31	C–C / C–H
**Powder with etching**	C 1s	285.41	4183.0	55.69	C–O / adventitious

Summary: layer composition and oxide thickness

Table S3 summarises the integrated Si^{4+} and Si^0 fractions, the corresponding intensity ratio used in the thickness calculation, and the resulting native-oxide thickness for each precursor. The post-RHEBM SiNPs@2-propynylamine product (main-text Figure 6, Table S1) is included as a fifth row to illustrate the disappearance of the fully oxidised Si^{4+} component upon mechanochemical functionalisation with 2-propynylamine.

Table S3. Layer composition and calculated SiO_2 thickness for the four silicon precursors and the post-RHEBM product.

Sample	Si^{4+} (%)	Si^0 (%)	I_Si^{4+} / I_Si^0	d_SiO_2 (nm)	Interpretation
**Wafer without etching**	84.92	15.08	5.63	**4.92**	Thick native SiO_2 layer; full Si^{4+} signature dominates.
**Powder without etching**	75.33	24.67	3.05	**3.64**	Mechanical fracturing exposes some Si^0 but oxide remains thick.
**Wafer with etching**	38.43	25.58	1.50	**2.38**	Etching partially removes oxide; sub-oxide component (~36 %) becomes visible.
**Powder with etching**	21.20	78.80	0.27	**0.62**	Strong oxide removal due to high surface area; Si^0 dominates the spectrum.
**SiNPs@2-propynylamine (post-RHEBM)***	**0.00**	9.10	—	—	Fully oxidised Si^{4+} absent. Surface dominated by Si–C / Si^0 (9.10 %) + sub-oxides Si^+/Si^{2+} (17.96 %) + Si^{3+} (72.94 %).

* Values for SiNPs@2-propynylamine are reproduced from Table S1 of the Supporting Information for comparative purposes; the Si 2p deconvolution of the post-RHEBM product is shown in main-text Figure 6.

Summary of the oxide-thickness trend

The corrected XPS analysis demonstrates a monotonic decrease in native-oxide thickness across the processing sequence:

Wafer without etching > Powder without etching > Wafer with etching > Powder with etching

4.92 nm > 3.64 nm > 2.38 nm > 0.62 nm

This trend is chemically reasonable: chemical etching decreases the native oxide contribution in both forms, and the effect is most pronounced for the powder because of the higher accessible surface area. The strong oxide reduction observed for the etched powder (≈87 % relative to the as-received wafer) explains why the

RHEBM step can reach the fully reduced/sub-oxide Si configuration observed in the SiNPs@2-propynylamine product, despite the relatively mild mechanochemical conditions used.

The Control Studies Confirmed that the Simultaneous Presence of Silicon and Amine Ligand is Essential for AuNP Formation

Table S4. Summary of the control experiments testing which components are required for interfacial Au^{3+} reduction and plasmonic AuNP formation (biphasic hexane–water system, 20 °C, 60 min). UV–vis spectra and photographs (10 and 60 min) are shown for each control.

C		UV–Vis	Photo (10 minutes-60 minutes)
1	Silicon, no ligand — bare Si does not reduce Au^{3+}	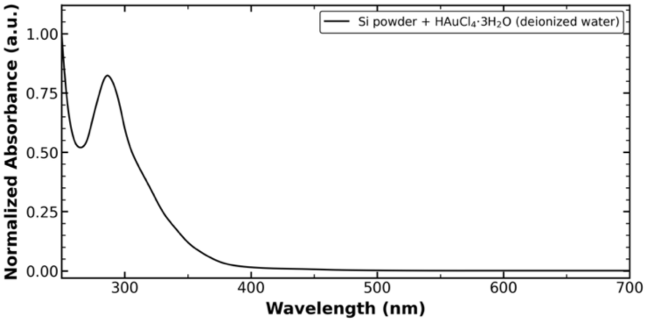	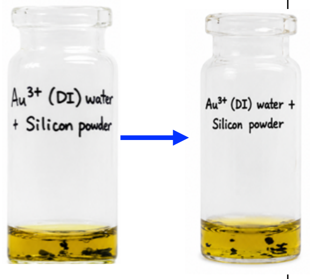
2	Free ligand, no Si — ligand alone not a reductant	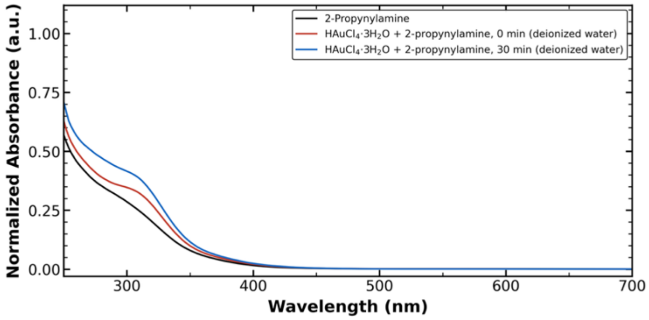	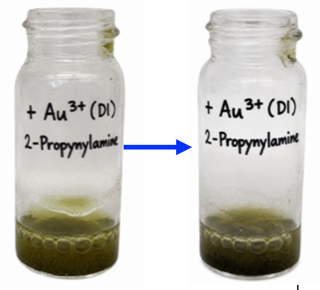
3	SiO_2@aminosilane — amine on insulator insufficient	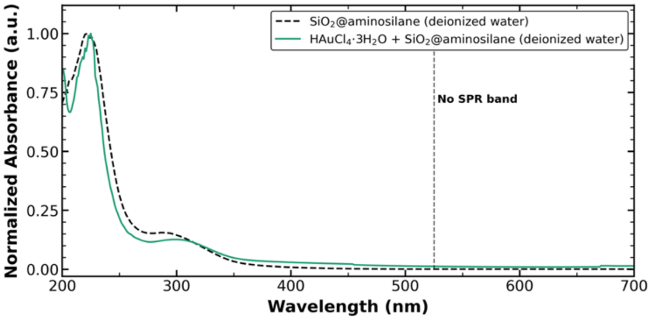	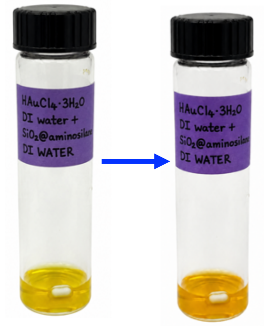

4	SiNPs, non-amine ligand — surface amine required	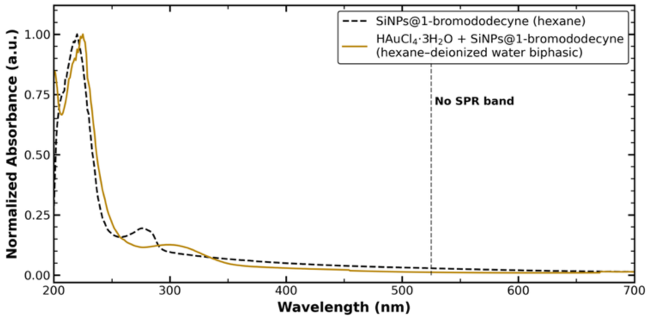	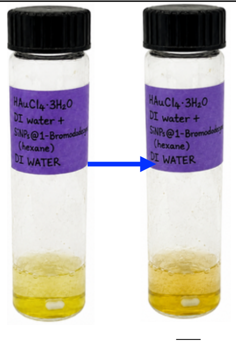
5	Tertiary amine (pargyline) — primary amine (N–H) required	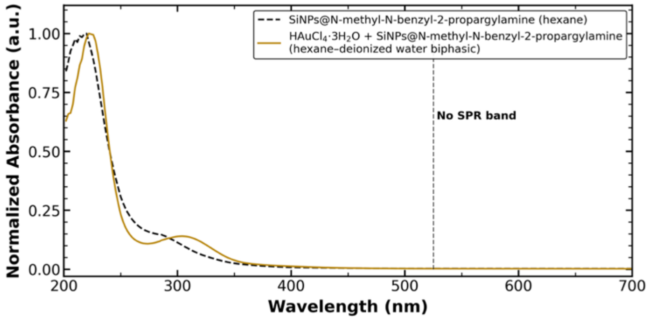	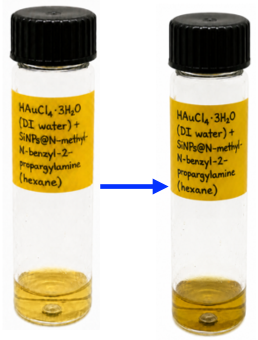
6	Si(amorphous) NPs@2-propynylamine	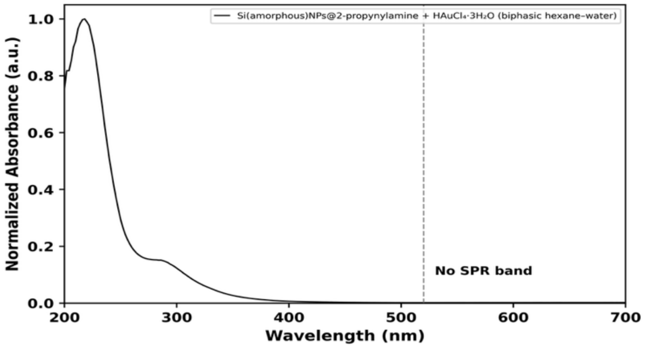	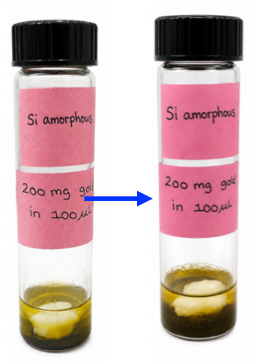

These control studies demonstrate that AuNP formation is governed by a cooperative mechanism requiring both silicon and a primary amine ligand. The primary amine serves as a coordination site for Au^{3+} ions, while the silicon surface provides the electronic environment necessary for their reduction. Neither component alone, nor alternative surface chemistries lacking the primary amine functionality, produced detectable AuNPs. These findings support a synergistic silicon–amine mechanism in which Au^{3+} adsorption, electron transfer, and nanoparticle nucleation occur at the functionalized silicon interface

Quantitative TEM Morphology Analysis of AuNPs@SiNPs@2-propynylamine

To support the claim of a mixed nanoparticle population, a systematic morphology analysis was performed on 12 representative TEM micrographs covering reaction times from 5 to 60 min. A total of 652 individual particles were classified into seven morphological categories using ImageJ-based contour analysis (see Methods).

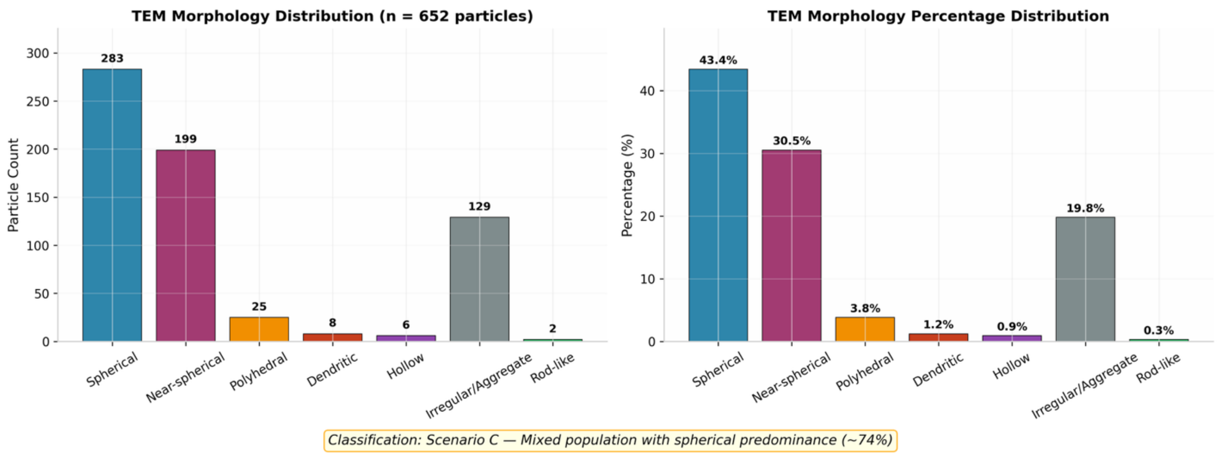

Figure S6. *Quantitative TEM morphology analysis of AuNPs@SiNPs@2-propynylamine. Particles (n = 652) were systematically counted across 12 representative micrographs covering reaction times from 5 to 60 min, and classified into seven morphological categories. The population is dominated by spherical (43.4%) and near-spherical (30.5%) nanoparticles, with polyhedral (3.8%), dendritic (1.2%), hollow (0.9%), irregular/aggregate (19.8%), and rod-like (0.3%) morphologies present as minority species. The classification corresponds to Scenario C of a mixed population with spherical predominance (~74%).*

Table S5. Per-image breakdown of the morphology counting analysis (n = 652 particles, 12 micrographs).

TEM Image	Time	Spherical (%)	Near-sph (%)	Polyhedral (%)	Dendritic (%)	Hollow (%)	Irreg/Agg (%)	Rod-like (%)	n
032	60 min	45.8	26.5	9.6	0.0	0.0	18.1	0.0	83
033	60 min	44.2	29.5	5.3	0.0	2.1	18.9	0.0	95
038	60 min	44.4	27.8	11.1	0.0	0.0	16.7	0.0	18
040	60 min	38.7	25.8	19.4	0.0	0.0	16.1	0.0	31
041	60 min	55.2	31.0	0.0	0.0	0.0	13.8	0.0	58
046	60 min	46.4	33.9	0.0	0.0	0.0	19.6	0.0	112
058	50 min	0.0	66.7	33.3	0.0	0.0	0.0	0.0	3
067	10 min	46.7	26.7	0.0	0.0	6.7	20.0	0.0	60
069	60 min	22.2	33.3	22.2	0.0	0.0	11.1	11.1	9

071	60 min	37.5	40.6	0.0	0.0	0.0	21.9	0.0	128
079	5 min	39.1	10.9	0.0	17.4	0.0	32.6	0.0	46
080	60 min	33.3	22.2	11.1	0.0	0.0	22.2	11.1	9
**TOTAL**	**All**	**43.4**	**30.5**	**3.8**	**1.2**	**0.9**	**19.8**	**0.3**	**652**

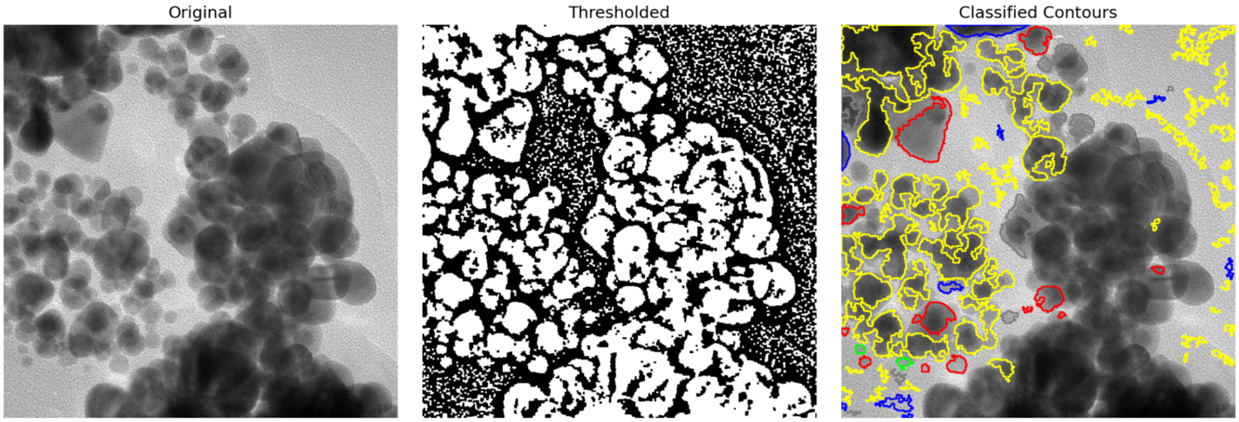

Figure S7. *Representative example of the morphology classification protocol applied to TEM micrographs. (Left) Original TEM image. (Center) Binary thresholding to isolate gold nanoparticles from the background. (Right) Classified contours overlaid on the original image, with colors corresponding to morphological categories: yellow = irregular/aggregate, red = polyhedral, blue = near-spherical, green = rod-like. Particles with areas below the detection threshold were excluded from the count.*

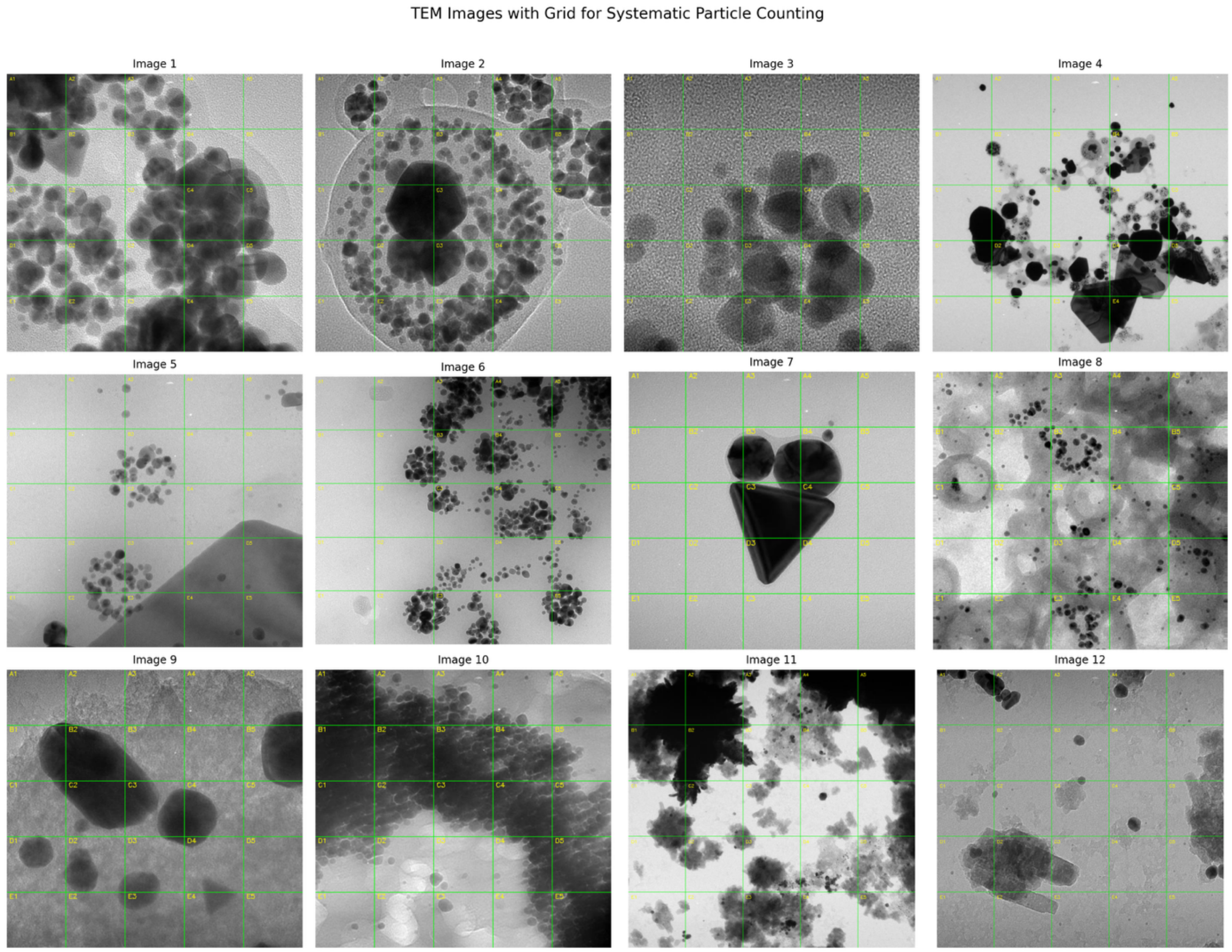

Figure S8. *TEM micrographs used for systematic morphology counting, with counting grid overlay. The 5×5 grid was applied to each of the 12 representative images to ensure systematic and unbiased particle classification across all fields of view (n = 652 particles total).*

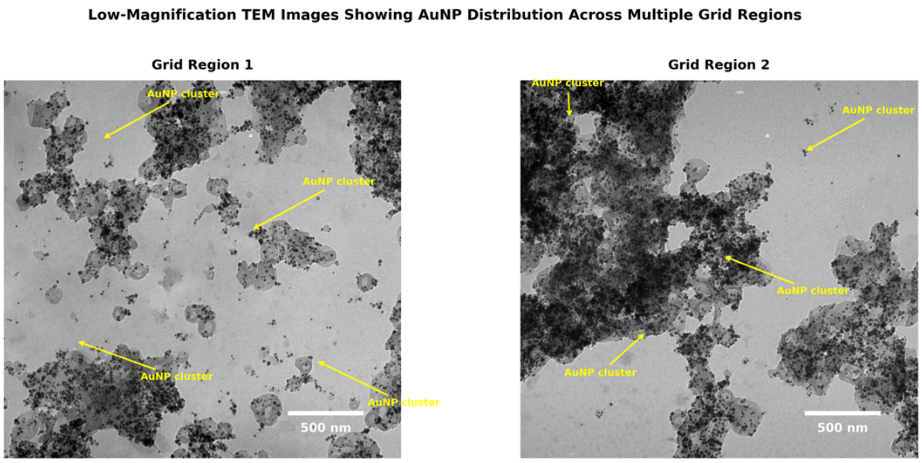

Figure S9. *Low-magnification TEM micrographs of AuNPs@SiNPs@2-propynylamine acquired from two distinct regions of the carbon-coated copper grid (500 nm scale bar). Panel (a) shows dispersed AuNP clusters distributed across an extended grid area (Region 1), while panel (b) reveals a dense interconnected network of AuNPs from a different grid location (Region 2). The distinct spatial patterns and consistent presence of AuNPs across multiple grid regions confirm that the nanoparticles are not confined to a sing*

Dynamic light scattering (DLS) of SiNPs@2-propynylamine and AuNPs@SiNPs@2-propynylamine

Dynamic light scattering (DLS) was employed as a complementary, ensemble-level size probe for the AuNPs@SiNPs@2-propynylamine hybrid system. The intensity-weighted hydrodynamic diameter (D_h) of the hybrid was 165 ± 12 nm with a polydispersity index of 24.5% and a coefficient of variation of 7.1% across twelve sub-measurements.

The distributions are weakly bimodal, with a minor sub-population near 10 nm (consistent with individual SiNPs/AuNPs) coexisting with a dominant population of a few hundred nanometres (consistent with hybrid assemblies). These ensemble values are larger than the primary particle sizes resolved by TEM (mean 3.7 ± 1.1 nm for SiNPs, Figure S3), as expected for an intensity-weighted technique that is strongly biased toward larger scatterers; the DLS data therefore corroborate, qualitatively, the broad size distribution and partial aggregation observed by TEM rather than providing a precise primary particle size.

Table S6 Compares the principal colloidal parameters of the SiNPs precursor and the AuNPs@SiNPs hybrid. Values are mean ± SD of the valid intensity-weighted sub-measurements. Only parameters reliably determined for both samples are listed here; the full set of distribution percentiles for the hybrid is given in Table S3. ζ-potential was not determined (these acquisitions measure size by back-scatter, not electrophoretic mobility).

Table S6. Principal colloidal parameters for the SiNPs precursor (back-scatter 175°) and the AuNPs@SiNPs@2-propynylamine hybrid (back-scatter 175°).

Parameter	SiNPs precursor (175°)	AuNPs@SiNPs hybrid (175°)
**D_h (nm)**	16 ± 5	165 ± 12
**PDI (%)**	25.6 ± 3.4	24.5 ± 1.7
**Mode (nm)**	~50	174
**Detection angle**	175° (back-scatter)	175° (back-scatter)
**n (valid)**	12	12

Figure S10 presents the full DLS dataset in four panels: the intensity-weighted size distributions of the SiNPs@2-propynylamine precursor (A) and the AuNPs@SiNPs@2-propynylamine hybrid (B), their overlay on a common axis (C), and a direct comparison of hydrodynamic diameter and polydispersity index (D). The precursor is centred near 16 nm, whereas the hybrid shifts to ~165 nm, evidencing substantial assembly and growth following interfacial gold reduction and deposition, while the polydispersity index remains essentially unchanged (~25%).

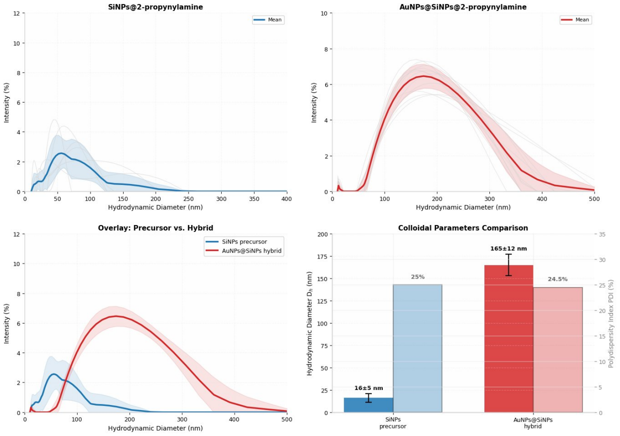

Figure S10. *DLS of the SiNPs precursor and the AuNPs@SiNPs hybrid. (A) Intensity-weighted size distribution of SiNPs@2-propynylamine (back-scatter 175°); thin grey traces are individual sub-measurements, the bold line the mean. (B) Intensity-weighted size distribution of the AuNPs@SiNPs@2-propynylamine hybrid (back-scatter 175°). (C) Overlay of both mean distributions, showing the size shift from precursor (blue)*

to hybrid (red). (D) Comparison of hydrodynamic diameter D_h (solid bars, left axis) and polydispersity index PDI (faded bars, right axis) for the two systems.

Figure S11 provides additional detail for the AuNPs@SiNPs@2-propynylamine hybrid. The autocorrelation function (A) shows a smooth single decay, confirming a good-quality measurement. The number-weighted distribution (B) is dominated by a small population near 8 nm, and the comparison of intensity-, volume- and number-weighted distributions (C) shows that the large ~165 nm population, which dominates the intensity-weighted signal, contributes negligibly on a number basis — consistent with a minor fraction of larger assemblies superimposed on a numerically dominant small-particle population. The cumulative (undersize) distribution (D) gives the percentile diameters D10, D50 and D90.

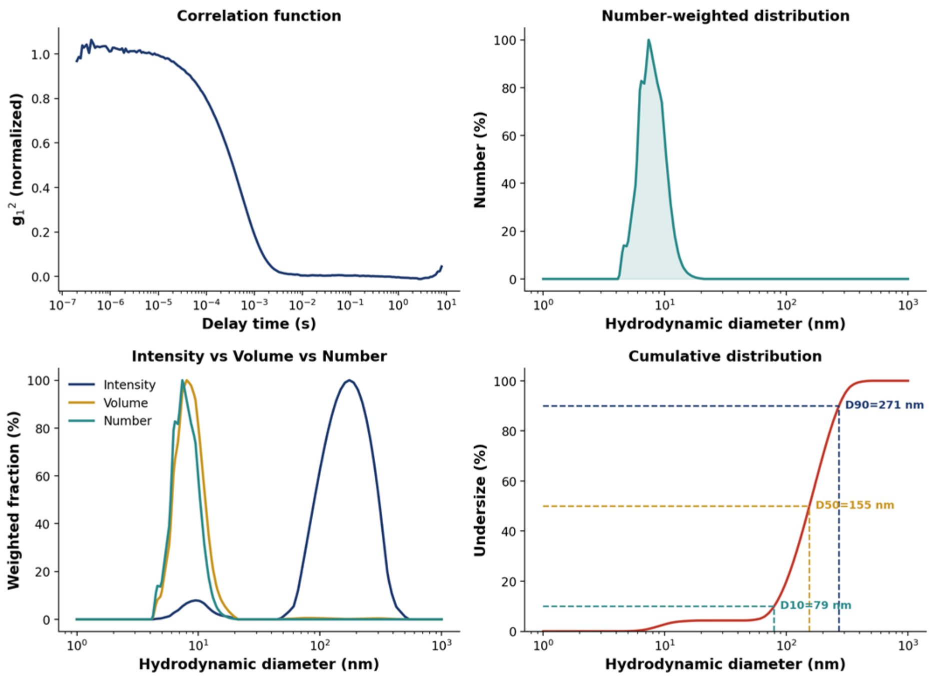

***Figure S11**. Additional DLS analysis of the AuNPs@SiNPs@2-propynylamine hybrid (back-scatter 175°). (A) Normalized autocorrelation function (g_1^2) versus delay time. (B) Number-weighted size distribution. (C) Overlay of intensity-, volume- and number-weighted distributions, illustrating how the larger assemblies dominate the intensity signal but are a minor fraction by number. (D) Cumulative (undersize) intensity-weighted distribution with the D10, D50 and D90 percentile diameters indicated.*

FTIR Peak Assignments for Free 2-Propynylamine, SiNPs@2-Propynylamine, and AuNPs@SiNPs@2-Propynylamine

Fourier-transform infrared (FTIR) spectroscopy was employed to confirm the surface functionalization and chemical interactions between 2-propynylamine, silicon nanoparticles (SiNPs), and gold–silica hybrid systems (AuNPs@SiNPs). The spectra of free 2-propynylamine, SiNPs@2-propynylamine, and AuNPs@SiNPs@2-propynylamine were compared to identify characteristic vibrational modes associated with the alkyne and amine functional groups, as well as shifts indicative of bonding with the silica surface and gold species.

Table S6 summarizes the observed peak positions, their corresponding literature values, and mode assignments. The presence and displacement of N–H, C≡C, and Si–O stretching vibrations confirm successful ligand attachment and the formation of hybrid nanostructures with distinct surface environments.

Table S7. FTIR peak assignments for free 2-propynylamine (red), SiNPs@2-propynylamine (blue) and AuNPs@SiNPs@2-propynylamine (green).

Assignment	Literature (cm^{-1})	Free 2-PA	SiNPs@2-PA	AuNPs@SiNPs @2-PA
N–H stretching	3365, 3330	3368, 3281	3345, 3275	3330, 3260
C–H (CH_2) stretching	2940	2926, 2850	2898, 2962	2918, 2948
C≡C stretching	2120–2130	2104	—	—
N–H bending (scissoring)	1640–1600	1591	1544	1604
CH_2 scissoring	1450	1437	1413	1415
Si–C stretching (overlap with Si–O–C)	1240–1260	—	~1251	—[1]
CH_2 wagging	1340	1384, 1342	1328	1326
Si–O stretching (oxidized Si surface)	1040–1080	—	1087[2]	1047[2]
C–N stretching	1070–1050	1064	1087	1047
N–H wagging	930–950	940	1004	—
C–C–C skeletal / propargyl chain mode	800–870	840	—	—
Si–C bending (rocking)	770–800	—	788	~790
Si–O bending (silanol groups)	690–720	—	—	698
C≡C–H bending	630–640	630	—	—
CCN deformation	530–540	534	—	—

[1]In the AuNPs@SiNPs@2-propynylamine spectrum the Si–C stretching is masked by the Si–O–Si asymmetric stretching band (~1080 cm^{-1}), which intensifies during AuNP deposition due to surface re-oxidation. Si–C bending at ~790 cm^{-1} is visible in both SiNPs and AuNPs spectra and is therefore the primary diagnostic for Si–C bond preservation in the hybrid

[2]The 1087 and 1047 cm^{-1} values are reported in both the C–N stretching and the Si–O stretching rows because the two modes overlap in this region and cannot be resolved into separate components in our spectra. The combined band is therefore listed under both assignments to acknowledge the contribution of each mode

Binding Energy Positions, FWHM, and Chemical Assignments from XPS Analysis of Gold, Silicon, Carbon, and Oxygen

High-resolution XPS spectra were collected to evaluate the chemical environment of the Au–Si hybrid interface. The deconvoluted spectra reveal characteristic peaks corresponding to metallic and oxidized gold species (Au^0, Au^+, Au^{3+}), as well as silicon in multiple oxidation states (Si^0, Si^+, Si^{2+}, Si^{4+}). Confirming the coexistence of reduced and partially oxidized domains.

The C 1s and O 1s regions exhibit peaks attributable to C–C, C–O, C=O, Si–C, and Si–O–C environments, providing evidence of covalent bonding between the silicon surface and the organic moieties introduced during functionalization. This suggests a chemically integrated Au–Si interface rather than simple physical adsorption.

Table S8. Binding Energy and Peak Area Parameters for Gold Components in the Au–Si Hybrid Material.

**Component**	**Binding Energy (eV)**	**% Area**	**Assignment**
Au $4f_{7/2}$ (Au^0)	84.28	47.38	Metallic Au core ($4f_{5/2}$ partner at 87.77 eV)
Au $4f_{7/2}$ (Au^{3+})	86.35	6.51	Oxidized Au (Au–O species, surface oxidation)
Au $4f_{5/2}$ (Au^{3+})	90.10	5.70	Partner of Au^{3+} doublet ($\Delta \approx$ 3.76 eV)
Low-BE tail / asymmetry	81.35	7.62	Inelastic tail of Au^0 (signal extension, not chemical state)

Partial surface oxidation and ligand-mediated interactions are responsible for stabilizing the hybrid structure, consistent with the spectroscopic and visual observations reported in the main text.

A summary of the peak fitting parameters, including binding energy (Position, eV)**,** FWHM**,** peak area**,** relative percentage (% Area), and chemical assignment is provided in Tables S2–S4 for gold, silicon, carbon, and oxygen, respectively.

Table S9. Binding Energy, and Peak Area Parameters for Silicon Components in the Au–Si Hybrid Material.

**Component**	**Binding Energy (eV)**	**% Area**	**Assignment**
Si $2p_{3/2}$ (Si^{2+} – Si^{3+})	100.95	66.87	Sub-oxide species (Si–O, Si–O–C, Si–OH)
Si $2p_{3/2}$ (Si^0)	99.26	30.15	Elemental Si / interfacial Si–C or Si–Au (silicon core)
Si $2p_{3/2}$ (Si^{4+})	103.23	2.97	SiO_2 / fully oxidized silicon (Si–O–Si network)

Table S10. Binding Energy and Peak Area Parameters for Oxygen Components in the Au–Si Hybrid Material.

**Component**	**Binding Energy (eV)**	**% Area**	**Assignment**
O 1s (Si–O / Si–O–C)	531.93	86.64	O bound to Si and C (Si–O–Si, Si–O–C)
O 1s (Au–O / interfacial oxygen)	529.29	13.36	O associated with Au–O or Au_2O_3-like species at the Au–Si interface

Table S11. Binding Energy and Peak Area Parameters for Carbon Components in the Au–Si Hybrid Material.

**Component**	**Binding Energy (eV)**	**% Area**	**Assignment**
C 1s (C–C/C–H, sp^2)	284.395	53.71	Hydrocarbon backbone / adventitious carbon
C 1s (C–C/C–H, sp^3)	285.028	34.61	Aliphatic carbon; tiny C–N/C–O contribution possible
C 1s (Si–C)	282.056	11.68	Interfacial carbide-like Si–C

XPS characterisation of the precursors and the nanoparticles

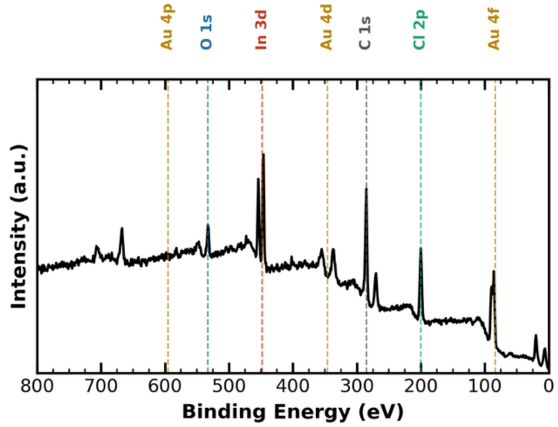

(a) $HAuCl_4 \cdot 3H_2O$

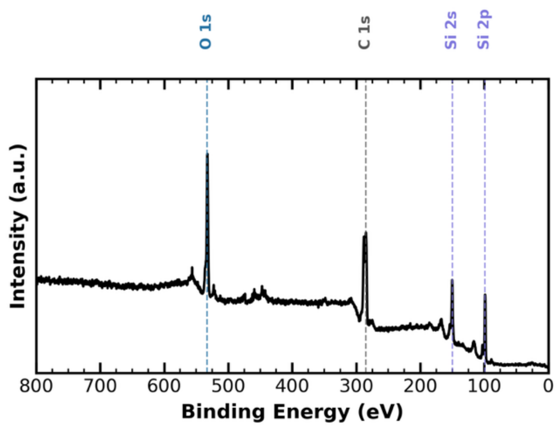

(b) Si wafer

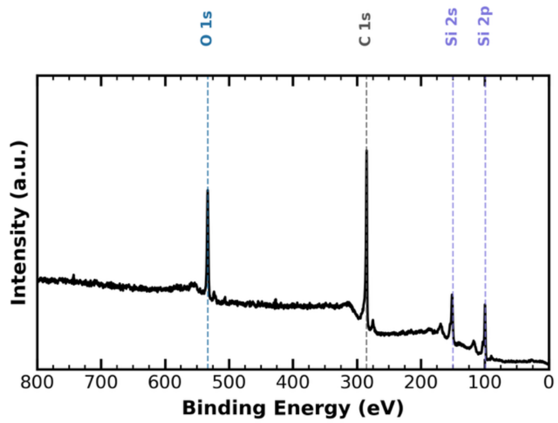

(c) Si powder

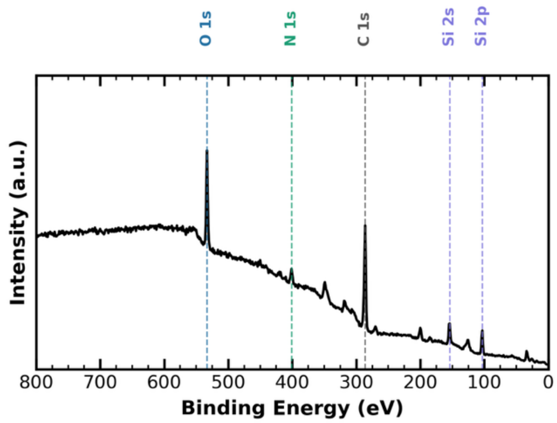

(d) SiNPs@2-propynylamine

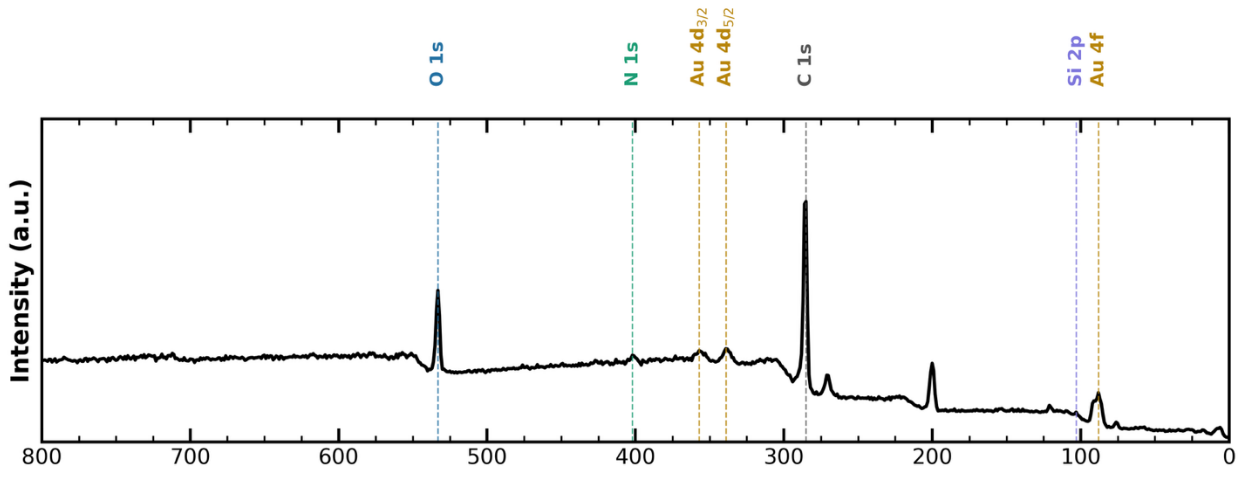

(e) AuNPs@SiNPs@2-propynylamine

Figure S12. *Survey XPS spectra of all characterized materials collected under identical acquisition conditions (Al Kα, 1486.6 eV; pass energy 200 eV; spot size 400 μm): (a) chloroauric acid trihydrate ($HAuCl_4{\cdot}3H_2O$) reference, showing Au 4f, Cl 2p, Au 4d, In 3d (indium foil substrate), O 1s, and Au 4p signals; (b) as-received Si wafer, showing Si 2p, Si 2s, C 1s, and O 1s consistent with a native oxide-covered silicon surface; (c) Si powder, displaying an elemental composition analogous to the wafer with a comparably thick native oxide; (d) SiNPs@2-propynylamine, where the appearance of an N 1s signal confirms successful surface functionalization with the alkynylamine ligand; and (e) AuNPs@SiNPs@2-propynylamine hybrid, displaying Au 4f and Au 4d signals alongside Si 2p, N 1s, and O 1s, consistent with gold nanoparticle deposition on the functionalized silicon surface. Adventitious carbon (C 1s, ~285 eV) is present in all samples. Atomic compositions derived from survey spectra are consistent with the high-resolution data reported in Tables S1 and S3–S6.*

XPS characterisation of the silicon precursors

Four silicon precursor states were characterized by high-resolution X-ray photoelectron spectroscopy (XPS) prior to reactive high-energy ball milling (RHEBM): (a) the as-received single-crystal wafer, (b) the mechanically fractured powder before etching, (c) the wafer after chemical etching, and (d) the etched powder, which is the precursor that enters the RHEBM reaction with 2-propynylamine. All four samples were measured under identical conditions on the Thermo Fisher Scientific Nexsa G2 system (Al Kα, 1486.6 eV, normal emission) described in the main-text Methods section. The Si 2p, O 1s and C 1s high-resolution spectra are shown in Figure S6.

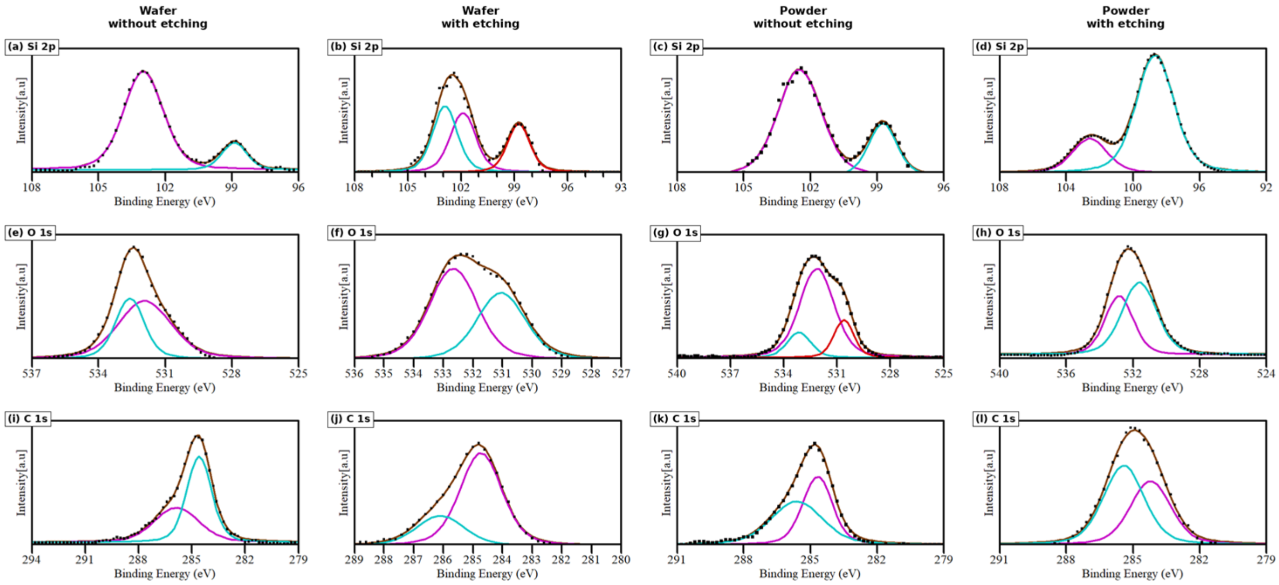

Figure S13. *High-resolution XPS spectra of the four silicon precursor states. Columns correspond to the four samples: wafer without etching, wafer with etching, powder without etching, and powder with etching. Rows show the Si 2p, O 1s and C 1s regions. Panels (a)–(d) Si 2p region: the Si^{4+} component near 103 eV (magenta) and the Si^{0} component near 99 eV (cyan) progressively change in their relative intensity from sample (a) to sample (d). The etched wafer (b) shows an additional sub-oxide component (red) at ~101.9 eV. Panels (e)–(h) O 1s region. Panels (i)–(l) C 1s region, dominated by adventitious carbon contributions in all four samples.*

References :

1. J. M. Hill, D. G. Royce, C. S. Fadley, L. F. Wagner and L. B. Grunzhan, *J. Vac. Sci. Technol.*, 1976, **12**, 324-328, DOI: 10.1116/1.568841.
2. F. J. Himpsel, F. R. McFeely, A. Taleb-Ibrahimi, J. A. Yarmoff and G. Hollinger, *Phys. Rev. B*, 1988, **38**, 6084-6096, DOI: 10.1103/PhysRevB.38.6084.
3. B. R. Strohmeier, *Surf. Interface Anal.*, 1990, **15**, 51-56, DOI: 10.1002/sia.740150107.
